# Reproductive tract extracellular vesicles are sufficient to transmit intergenerational stress and program neurodevelopment

**DOI:** 10.1038/s41467-020-15305-w

**Published:** 2020-03-20

**Authors:** Jennifer C. Chan, Christopher P. Morgan, N. Adrian Leu, Amol Shetty, Yasmine M. Cisse, Bridget M. Nugent, Kathleen E. Morrison, Eldin Jašarević, Weiliang Huang, Nickole Kanyuch, Ali B. Rodgers, Natarajan V. Bhanu, Dara S. Berger, Benjamin A. Garcia, Seth Ament, Maureen Kane, C. Neill Epperson, Tracy L. Bale

**Affiliations:** 1 Department of Biomedical Sciences, School of Veterinary Medicine and Perelman School of Medicine, University of Pennsylvania, Philadelphia, PA 19104 USA; 2 Department of Pharmacology and Center for Epigenetic Research in Child Health and Brain Development, University of Maryland School of Medicine, Baltimore, MD 21201 USA; 3 Institute for Genome Sciences, University of Maryland School of Medicine, Baltimore, MD 21201 USA; 4 Department of Pharmaceutical Science, University of Maryland School of Pharmacy, Baltimore, MD 21201 USA; 5 Epigenetics Institute, Department of Biochemistry and Biophysics, Perelman School of Medicine, University of Pennsylvania, Philadelphia, PA 19104 USA; 6 Department of Obstetrics and Gynecology, Division of Reproductive Endocrinology and Infertility, Perelman School of Medicine, University of Pennsylvania, Philadelphia, PA 19104 USA; 7 Department of Psychiatry, Perelman School of Medicine, University of Pennsylvania, Philadelphia, PA 19104 USA

**Keywords:** Epigenetic memory, Epigenetics, Epigenetics, miRNAs, Development of the nervous system

## Abstract

Extracellular vesicles (EVs) are a unique mode of intercellular communication capable of incredible specificity in transmitting signals involved in cellular function, including germ cell maturation. Spermatogenesis occurs in the testes, behind a protective barrier to ensure safeguarding of germline DNA from environmental insults. Following DNA compaction, further sperm maturation occurs in the epididymis. Here, we report reproductive tract EVs transmit information regarding stress in the paternal environment to sperm, potentially altering fetal development. Using intracytoplasmic sperm injection, we found that sperm incubated with EVs collected from stress-treated epididymal epithelial cells produced offspring with altered neurodevelopment and adult stress reactivity. Proteomic and transcriptomic assessment of these EVs showed dramatic changes in protein and miRNA content long after stress treatment had ended, supporting a lasting programmatic change in response to chronic stress. Thus, EVs as a normal process in sperm maturation, can also perform roles in intergenerational transmission of paternal environmental experience.

## Abstract (web summary)

Evidence for sperm small noncoding RNA-mediated intergenerational transmission implies communication from responsive somatic cells to sperm. Here, authors show that epididymal cells alter extracellular vesicle cargo after stress exposure, to impact offspring neurodevelopment and stress reactivity.

## Introduction

The importance of the preconception environment in shaping post-fertilization development, initially suggested by epidemiological studies in humans whereby parental environmental exposures can have a long-lasting impact on the next generation, has been documented independently in numerous model organisms^1–26^. Changes detected in sperm, including small noncoding RNA populations, are suggested mediators between paternal exposures and the programming of offspring development^11,25,27–33^. However, as the post-spermatogenic germ cell is transcriptionally inert and its DNA compacted to protect the germline^34^, somatic mechanisms would need to exist whereby the environment can exert significant and lasting effects on sperm content and paternally-derived post-fertilization outcomes. Critical sperm maturation processes occur in the tubules of the caput epididymis where sperm receive somatically-derived signals. We hypothesized that chronic challenges to organismal homeostasis become encoded in somatic tissues outside of the testicular environment, specifically in caput epididymal epithelial cells (EECs), and are then communicated to sperm during their post-spermatogenic maturation. Extracellular vesicles (EVs) are a mode of intercellular communication, enabling the specific delivery of bioactive cargo, including proteins, lipids, and small noncoding RNAs from a donor cell to change the function and/or content of a recipient cell^35^. The field of reproductive biology has clearly demonstrated the involvement of EVs in transferring key maturational signals from caput EECs to sperm within the epididymal lumen that are required for normal post-spermatogenic development^36–38^. The co-opting of this intercellular communication mechanism is an efficient strategy to transfer relevant signals regarding the dynamic paternal preconception environment to alter the course of embryo development at fertilization. The evidence for the existence of this male intergenerational transmission has been suggested by a growing number of studies in rodents chronically challenged with stress, dietary constraints, or drugs^9,12,14,15,17–20,26^. However, evidence for the somatic-to-germline delivery of such a signal, the mechanism underlying its post-exposure persistence, or a causal link between changes to EV cargo and embryo development remains unclear.

## Results

### Sperm miRNA and intergenerational transmission after stress

**Fig. 1:**
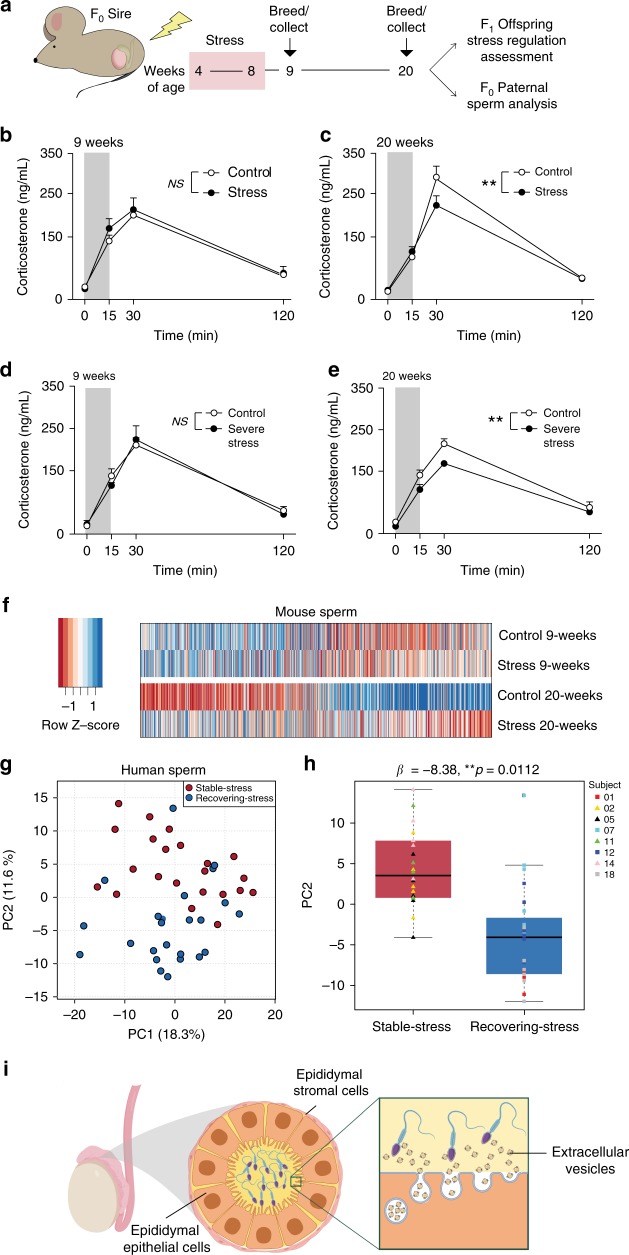
Stress dynamics impact intergenerational transmission and sperm miRNA content. **a** F_0_ breeding time course with periods of stress (4 weeks) and recovery (1 or 12 weeks after stress) for sperm and F_1_ offspring assessment. **b** Transmission of a stress phenotype in the corticosterone response to an acute 15-min restraint (gray bar) did not occur for offspring conceived at 9 weeks (two-way rmANOVA, time (F(3,39) = 88.07, *p* = 2.0375 × 10^−17^), paternal treatment (F(1,13) = 0.5528, *NS* *p* = 0.4704, *N* = 7–8 offspring/paternal treatment). **c** Offspring conceived at 20 weeks had altered stress reactivity (two-way rmANOVA, interaction of paternal treatment × time (F(3,42) = 3.718, *p* = 0.0185), time (F(3,42) = 138.5, *p* = 8.3427 × 10^−22^), paternal treatment (F(1,14) = 1.179, *p* = 0.2959)), with significant reduction in the maximal rise of corticosterone at 30 min (Bonferroni’s post hoc test, *t*(56) = 3.29, **adjusted *p* = 0.0069, *N* = 8 offspring/paternal treatment). **d** Doubling F_0_ stress (severe stress) did not transmit a stress phenotype for offspring conceived at 9 weeks (two-way rmANOVA, time (F(3,39)=92.52, *p* = 8.8203 × 10^−18^; paternal treatment F(1,13)=0.0667, *NS* *p* = 0.8002, *N* = 7–9 offspring/paternal treatment). **e** Severe stress transmission altered stress reactivity for offspring conceived at 20 weeks (two-way rmANOVA, interaction of paternal treatment × time (F(3,30) = 2.600, *p* = 0.0705), time (F(3,30) = 173.5, *p* = 4.4839 × 10^−19^; paternal treatment (F(1,10) = 8.199, *p* = 0.0169), with significant reduction in the maximal rise of corticosterone at 30 min (Bonferroni’s post hoc test, *t*(40) = 3.549, **adjusted *p* = 0.0040, *N* = 6 offspring/paternal treatment). Error bars represent mean ± SEM. **f** Expression patterns for all sperm miRNA detected by RNA sequencing as indicators of germ cell reprogramming, correlating with timing of phenotypic transmission. miRNA values were averaged within treatment groups (*N* = 6–8 samples/paternal treatment/time post-stress). **g** Examination of sperm miRNA dynamics and perceived stress in a human cohort. Principal component (PC) analysis of sperm based on all sequenced miRNA demonstrated the influence of perceived stress dynamics on sperm expression patterns, where PC2 largely segregated samples collected from ‘stable-stress’ (blue circles) and ‘recovering-stress’ subjects (red circles). *N* = 5–6 sperm samples/subject/stress phenotype group (see Methods). **h** Quantification showing significant association between a subject’s sample PC2 score and its stress phenotype group (regression model β-coefficient = −8.38, **p = 0.0112). Data are median with first and third quartiles (box) and top and bottom quartiles (whiskers) indicated. Individual data points are colored to denote subject. **i** Hypothesized model of maturing sperm interacting with extracellular vesicles within the caput epididymal lumen.

To investigate the somatic cell molecular mechanism underlying the lasting impact of environmental stress experience on intergenerational transmission, we developed a novel paradigm whereby paternal tissues were compared at a time point post-stress that was not able to transmit a phenotype to subsequent offspring with a time point where phenotypic transmission occurred and was multiple spermatogenic cycles after stress end, indicative of a lasting impact of stress. Therefore, while utilizing our previous chronic stress model as a premise for the current studies, we modified the post-stress breeding time points to address these mechanistic questions. We exposed male mice to 4 weeks of chronic stress between 4- and 8 weeks of age, and bred these animals to naive females either 1 week following the end of the stress exposure (at 9 weeks old) or 12 weeks following the end of stress (20 weeks old, Fig. 1a). Based on previous studies from our lab^10,30^, we assessed hypothalamic–pituitary–adrenal (HPA) stress axis dysregulation outcomes in adults. A phenotypic change was not detectable in offspring conceived 1 week following the end of stress (Fig. 1b). In contrast, offspring conceived 12 weeks following stress end presented with the predicted HPA axis phenotype^10^ (Fig. 1c). To confirm the absence of intergenerational transmission at 1 week was not due to an insufficient stress signal, we increased the severity of chronic stress the sires experienced over the same 4-week period and detected the same offspring outcomes, with no changes in HPA reactivity in offspring conceived 1 week after stress ended (Fig. 1d), but a significant change in offspring conceived 12 weeks after stress (Fig. 1e). These results support the hypothesis that a period of recovery after stress, regardless of the intensity of the experience, is important to produce a lasting intergenerational transmission of an HPA stress axis phenotype in offspring.

Next, to confirm an association between changes in sperm and timing of intergenerational transmission, we compared paternal sperm miRNA by small RNA sequencing, as recent studies in animal models and in humans have established that small noncoding RNA levels associated with sperm are dynamic in response to environmental perturbations^10,27,28,30–33,39,40^. While minor differences in sperm miRNA expression patterns were found between control and stress sires 1 week following stress end, we detected dramatic group differences 12 weeks after stress had terminated (Fig. 1f), including an effect of age (see below). Supplementary Data 1 contains each miRNA identified by small RNA sequencing and *p* values from differential expression analysis. Supplementary Data 2 contains statistics for small RNA-sequencing validation data.

To determine if such a dynamic state of sperm miRNA also exists in human sperm and whether a pattern of change could be related to prior stress state, we recruited men from a relatively homogenous and ‘normative’ population of University of Pennsylvania students. Subjects between the ages of 18 and 25 were screened and excluded for major medical illness, mental health diagnoses, and substance abuse. Following screening and baseline assessments, enrolled subjects returned monthly for 6 months to donate semen samples for sperm miRNA analysis. In addition, with each sample donation subjects completed psychological inventories, including the Perceived Stress Scale^41^, to assess their stress experience during the prior month (Supplementary Fig. 1a). This repeated measures design allowed us to perform within- and between-subjects comparisons over time to examine the impact of prior stress experience and recovery on sperm miRNA expression patterns. Specifically, to best align with outcomes detected from our mouse model, we sought to identify subsets of males who either (1) had experienced a period of elevated stress followed by an extended period of recovery (recovering-stress dynamic), or (2) showed little-to-no variation in stress levels over time (stable-stress dynamic).

Following recruitment screening, 18 males completed all requirements and donations for the study, though one individual (subject 11) failed to return for his final donation. Three subjects were excluded from analysis due to consistently poor sample quality. Baseline demographics and results from an Adverse Childhood Experiences (ACE) questionnaire and Spielberger State-Trait Anxiety Inventory (STAI) demonstrate the final study cohort (*N* = 15) was relatively homogeneous (Supplementary Data 3)^42,43^. In examining subject PSS scores reported over the 6 months (Supplementary Fig. 1b), we identified four individuals that fit our criteria for a recovering-stress dynamic, as characterized by a significant drop in PSS scores ≥10 over the course of the 6-month study^44^, and four subjects who had minimal-to-no variation in perceived stress over time (stable-stress state), regardless of their overall perceived stress level. Mature sperm was enriched from cryopreserved samples collected from these individuals at all time points and subjected to small RNA sequencing. Amazingly, from this small subset of human subjects, we identified broad shifts in miRNA expression resulting from differences in stress experience while taking into consideration individual variation over time (Supplementary Fig. 1c).

We then performed an unbiased multivariate PCA analysis to identify additional factors that distinguished sperm samples based on miRNA expression. This analysis demonstrated the extensive influence of prior stress experience and recovery on human sperm miRNA expression profiles (Fig. 1g). Specifically, principal component 2 (PC2) largely segregated samples collected from the stable-stress subjects from those of the recovering-stress subjects. To validate the relationship between principal component 2 and the stress-experience dynamic, we implemented a linear mixed effects model using subject as a random effect to account for repeated measures (Fig. 1h). This model showed there was a statistically significant association between a sample’s PC2 score and the stress phenotype of the subject it was collected from (regression model β coefficient = −8.38, *p* = 0.0112), demonstrating sperm miRNA levels continue to be dynamic during recovery from stressful experiences, generating distinct sperm miRNA profiles in both mice and humans.

Based on these data, we hypothesized that the caput EEC was responding to the previous extended period of stress and transmitting a changed signal to maturing sperm in the epididymal lumen via EVs (Fig. 1i, schematic created with BioRender). Given this long-term window post-stress, we postulated the involvement of somatic chromatin changes as a mode by which stress can be encoded in the epididymal epithelium long-term and impact sperm content. To align potential caput EEC changes to observed changes in mouse sperm miRNA, we performed histone post-translational modification (PTM) mass spectrometry on caput epididymal tissues from control and stress mice at 9 and 20 weeks of age, allowing the total identification of 306 histone PTMs (Supplementary Fig. 2a and Supplementary Data 4). Comparing the timing of stress effects on the caput epididymal histone signature between 9- and 20 weeks, an unbiased principal components analysis (PCA) of all histone PTMs clustered control and stress groups together at 9 weeks, but showed a dramatic effect of stress by 20 weeks (Supplementary Fig. 2c). Interestingly, the greatest variance (PC1) in both treatment groups appears to be driven predominantly by time (age), suggesting that the caput epididymal histone code matures with age and that stress has significantly disrupted this normal process, which aligns with the observed timing of sperm miRNA expression changes (Fig. 1f). Modeling the effects of age within control tissues using Random Forests analysis^45^, we demonstrate that these normally maturing histone PTMs are disrupted in the caput epididymis by prior stress exposure (Supplementary Fig. 2d–g), similar to our observations for sperm miRNA and age.

Caput EECs secrete EVs carrying bioactive cargo, including miRNA and protein, that are crucial for normal sperm maturation. Thus, we next assessed the timing of and changes in EV content by small RNA sequencing and proteomics from EEC-secreted EVs produced following stress treatment.

### Stress treatment impacts epididymal EV cargo

**Fig. 2:**
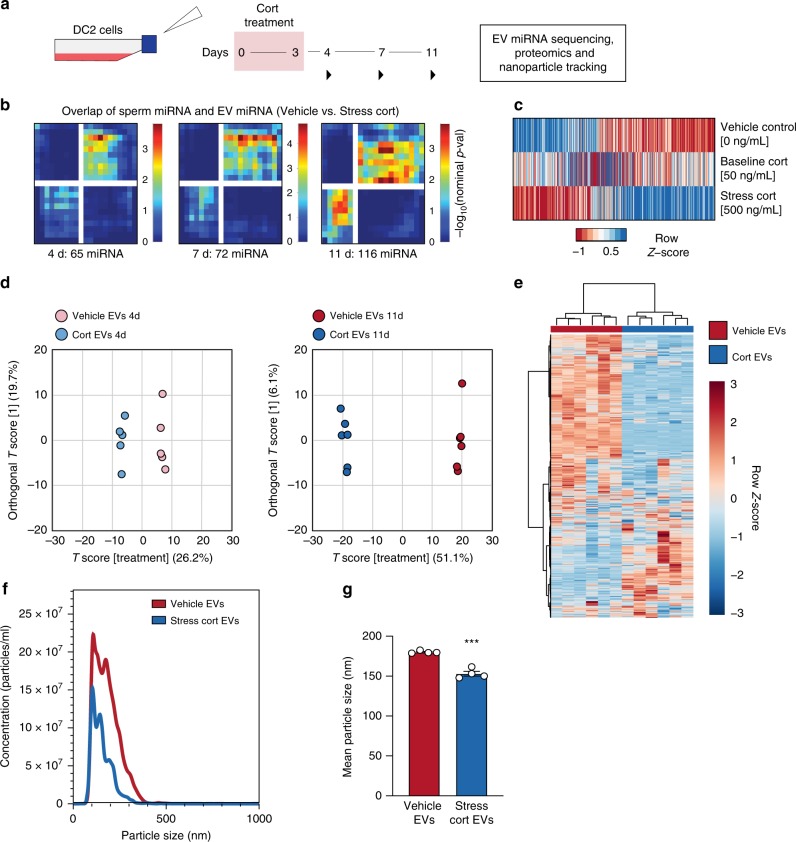
Stress recovery alters miRNA and protein content of epididymal epithelial cell-secreted extracellular vesicles (EVs). **a** Time course of corticosterone (Cort) treatment (3 days) and recovery (1, 4, or 8 days) in DC2 mouse caput epididymal epithelial cells, using physiologically-relevant concentrations for baseline (50 ng/mL) and stress (500 ng/mL) levels of corticosterone. Gray triangles indicate time points for EV collection for transcriptomic, proteomic, and size assessment. **b** Rank–rank hypergeometric overlap analysis was used on total sequenced miRNA. The differential expression profiles of sperm 12 weeks post-stress were plotted against the differential expression profiles of EVs collected 1, 4, or 8 days post-corticosterone treatment (left, middle, and right, respectively). Overlap data are plotted as sperm miRNA ratios increasing down the *y*-axis and EV miRNA ratios increasing left along the *x*-axis, with each pixel representing the −log_10_(nominal *p*-value) of overlapping miRNA via the hypergeometric distribution and color coding according to degree of significance (as shown). Each RRHO heatmap is divided into four quadrants, where the bottom-left and upper-right squares represent concordant miRNA changes in both models, quantified below each heatmap (*N* = 3–4 EVs/treatment/time, max −log_10_(*p*-value) = 5). **c** RNA-sequencing examination of all detectable miRNA from secreted DC2 EVs 8 days following stress levels of corticosterone treatment as indicators of changes in EV programming compared with vehicle treatment (*N* = 3–4 EVs/treatment). **d** Proteomic mass spectrometry comparison of secreted DC2 EV protein content analyzed by orthogonal partial least squares analysis show the impact of corticosterone programming, accounting for 26.2% of EV protein variation at 1 day after (left) and progressed to 51.1% at 8 days after treatment (right, *N* = 5–6 EVs/treatment/time). **e** Heatmap of total identified EV proteins by mass spectrometry 8 days post-corticosterone treatment compared with vehicle with hierarchical clustering of samples (*N* = 6 EVs/treatment). **f** Nanosight particle tracking identified a significant reduction in size distribution of the total population from secreted EVs 8 days post-corticosterone treatment compared with vehicle, plotted as average values across replicates (*N* = 4 EVs/treatment). **g** Quantification of Nanosight plots showing the mean size of EVs secreted by DC2 cells was significantly reduced 8 days following corticosterone treatment compared with vehicle (two-tailed unpaired Student’s *t*-test, *t*(6) = 8.68, ***p = 0.0001, *N* = 4 EVs/treatment). Error bars are SEM.

All mammalian tissues secrete diverse populations of EVs from multiple cell types, making the specific isolation and examination of a pure population of mouse caput EEC EVs from this heterogeneous tissue not possible with current technologies. Therefore, we developed an in vitro culture system using DC2 mouse caput EECs to determine the ability of the stress hormone, corticosterone, to program long-term changes in secreted EVs in a timeframe aligning with paternal stress exposure and recovery in vivo. To accurately model the timing of stress encoding in caput epididymal tissue from our in vivo conditions, we compared the effect of 3 days of chronic corticosterone treatment at concentrations relevant to mouse baseline levels (nadir) and stress levels at 1, 4, and 8 days following hormone treatment in DC2 cells (4, 7, and 11 days, respectively) (Fig. 2a). Isolated EVs were validated by both western blot analysis (Supplementary Fig. 3) and nanoparticle tracking (Nanosight). Analyses of small RNA sequencing on EV samples compared miRNA profiles to those identified from our sire sperm samples at 12 weeks of recovery from stress, when intergenerational transmission occurred. We used rank–rank hypergeometric overlap (RRHO) analyses to evaluate and quantify the extent of common miRNA changes detected between our sire sperm samples and DC2 EVs, allowing for threshold-free identification followed by quantification of statistically significant overlap of miRNA between datasets^46,47^. Moreover, using an advanced version of RRHO analysis, we were able to categorize these comparisons by directionality and concordance^46,47^. We identified patterns of increasing miRNA overlap over time following stress treatment, where the greatest degree of overlap was detected 8 days following hormone treatment (Fig. 2b and Supplementary Fig. 4). No doubt, the total complexity of sperm small noncoding RNA composition reflects additional interactions along the entirety of the reproductive tract, and therefore will not completely mirror the DC2 EV profile, as has been described^48,49^. We found significant overall changes in miRNA expression patterns from DC2 EVs 8 days following stress treatment compared with vehicle or baseline corticosterone treatment (Fig. 2c), and validated these observations with quantitative reverse-transcription-PCR (Supplementary Fig. 2b and Supplementary Data 2 and 5).

EVs also contain proteins critical to their cellular specificity and function. Using mass spectrometry to determine proteomic changes in DC2 EV cargo following corticosterone treatment as above, orthogonal partial least squares (OPLS) analysis showed that protein differences increased over time post treatment, where the variance in proteins attributable to corticosterone 1 day post treatment was 26.2% (Fig. 2d, left and Supplementary Fig. 5), but grew to 51.1% by 8 days post treatment (Fig. 2d, right and Supplementary Data 6). These dramatic differences in EV protein content 8 days following hormone treatment were apparent by hierarchical clustering of all detected proteins (Fig. 2e). Comparison of size distribution of DC2 EVs using nanoparticle tracking analysis showed a significant reduction in average vesicle size following stress hormone treatment (Fig. 2f, g), consistent with the changes found in the composition that may reflect EV production from different intracellular compartments and/or a more select population of EVs (e.g., exosomes vs. microvesicles).

To confirm tissue targeting specificity retention of in vitro generated DC2 EVs in vivo, we fluorescently labeled vehicle- and corticosterone-treated DC2 EVs with the near-infrared lipophilic DiR dye and injected 50 million EVs intravenously into naive male mice (Supplementary Fig. 6a). Twenty-four hours post-injection, tissues were removed and imaged to evaluate the bio-distribution of DC2 caput EEC EV targeting. As previously described for EVs from most other cellular sources, we observed substantial accumulation of EVs in the liver and spleen^50^. However, there was also selective accumulation of EVs within the reproductive tract, including the caput epididymis and testes, demonstrating retention of DC2 EEC-secreted EV targeting specificity (Supplementary Fig. 6b–c). Quantification of DiR dye showed no significant differences in the abundance of DC2 EV accumulation between treatment groups (Supplementary Fig. 6d), confirming miRNA and protein changes to DC2 EVs likely do not disrupt their targeting selectivity. These studies support our hypothesis that stress encoding in reproductive somatic cells occurs during stress recovery, and may be communicated to the germ cell through changes in EV bioactive cargo.

### ICSI with stress EEC EVs alters neurodevelopment

**Fig. 3:**
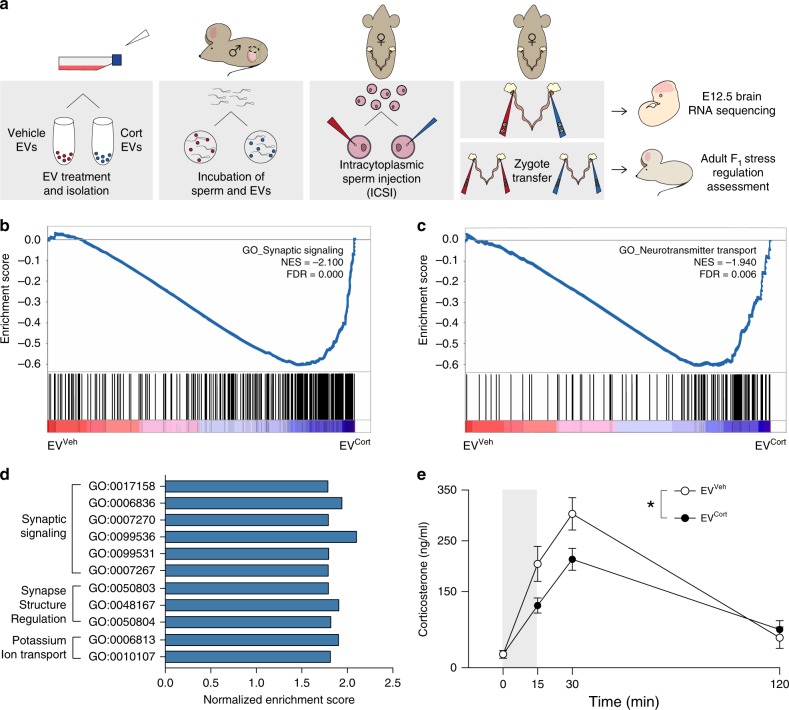
Intracytoplasmic sperm injection (ICSI) of sperm incubated with extracellular vesicles (EVs) secreted following stress recovery alter offspring neurodevelopment. **a** Schematic representation of ICSI paradigm using sperm incubated with vehicle (EV^Veh^) or corticosterone-treated (EV^Cort^) EVs, followed by microinjection into super-ovulated oocytes obtained from the same female donors. Zygotes from both EV-treatment groups were transferred into the designated right or left side of the same naive foster females to assess offspring neurodevelopment at mid-gestation (E12.5, top), or into separate recipient females for assessment of an F_1_ stress phenotype in adulthood (bottom). **b**, **c** Gene set enrichment analysis (GSEA) was used to analyze RNA-sequencing of E12.5 brains, with significant enrichment of gene sets related to (**b**) synaptic signaling and (**c**) neurotransmitter transport in embryos from EV^Cort^ sperm compared with EV^Veh^ sperm (*N* = 6 embryos/EV treatment, GSEA normalized enrichment score (NES) > |1.8|, FDR < 0.05). **d** The top three significant clusters of gene ontology (GO) terms enriched in EV^Cort^ E12.5 brains determined by GSEA and clustered under parent terms were related to synaptic signaling (*N* = 6 embryos/treatment, NES > |1.8| and FDR < 0.05). **e** EV^Cort^ adult F_1_ offspring showed the same phenotypic pattern of stress reactivity to an acute 15-min restraint (gray bar) as paternal stress F_1_ offspring, compared with EV^Veh^ F_1_ offspring (two-way rmANOVA, interaction of EV treatment × time F(3,21) = 8.480, *p* = 0.0007, time (F(3,21) = 122.6, *p* = 1.8125^−13^), EV treatment (F(1,7) = 1.868, *p* = 0.2139, *N* = 4–5 offspring/EV treatment), with a significant reduction in the maximal rise of corticosterone at 30-min (Bonferroni’s post hoc test, *t*(28) = 2.733, *adjusted *p* = 0.043). Error bars represent mean ± SEM.

To examine the ability of EEC EVs to transfer information regarding stress in the environment at fertilization, we utilized the assisted reproductive technology, intracytoplasmic sperm injection (ICSI). We extracted and purified caput epididymal sperm from naive adult male mice and divided sperm samples into two pools that were incubated with either previously vehicle-treated DC2 EVs (EV^Veh^ sperm) or corticosterone-treated DC2 EVs (EV^Cort^ sperm) collected at 8 days after treatment. ICSI was performed using oocytes harvested from the same donor females and microinjected with a single sperm, followed by embryo transfer into the same naive recipient female, into either the right or left side of the uterus based on EV-treatment group, such that both groups were represented within each uterus to control for the intrauterine environment. To determine the impact on fetal development, pregnant dams were sacrificed at mid-gestation (E12.5), and embryonic tissue was examined for transcriptional changes in brain and placental development using gene set enrichment analysis (GSEA) (Fig. 3a). GSEA analysis identifies groups of genes from specified gene lists that are statistically over-represented (enriched or depleted) in curated biological signatures thereby providing functional annotation of RNA-sequencing results. E12.5 developing brains derived from EV^Cort^ sperm showed significant changes in gene sets enriched for synaptic signaling and neurotransmitter transport (Fig. 3b, c), supporting important changes in neurodevelopment that may impact adult brain function. In examination of all significantly enriched gene sets determined by GSEA, using ClusterMaker2 to summarize redundant GO terms, we found that synaptic signaling encompassed 19.3% of gene sets enriched in the EV^Cort^ E12.5 brains, in addition to GO terms related to neuronal communication (Fig. 3d and Supplementary Data 7). 9.6% of gene sets depleted in EV^Cort^ brains were related to regulation of type 1 interferon production. Additional depleted gene sets included those related to blood and immune processes (Supplementary Fig. 7 and Supplementary Data 8). In our analyses of placental tissue, EV^Cort^ sperm also influenced the placental transcriptome, resulting in enrichment of inflammatory and immune responses and decreases in gene sets related to chromosomal and chromatin processes (Supplementary Fig. 8 and Supplementary Data 9), which may further impact brain development as the placenta contributes critical developmental signals to the fetus^51^.

To evaluate the causal role of reproductive tract EVs in transmitting an intergenerational signal related to stress in the paternal environment, and EV^Cort^-incubated sperm on reproducing our paternal stress offspring phenotype, we examined stress reactivity in adult offspring following ICSI of EV^Veh^- vs. EV^Cort^-incubated caput sperm in which embryos were transferred into recipient dams and allowed to develop normally. Indeed, an HPA axis assessment showed that EV^Cort^ offspring exhibited the same dysregulation in stress responsivity (Fig. 3e), recapitulating offspring outcomes from germline transmission of paternal stress. We also examined the growth trajectory of these offspring and found that increased body weights of EV^Cort^ offspring were detectable during a pubertal window of development, but normalized by early adulthood, and were not associated with abnormalities in any standard litter characteristics (Supplementary Fig. 9).

## Discussion

Debate as to the existence of evidence for intergenerational epigenetic inheritance in humans has recently intensified^52,53^. In the current studies, we demonstrated a causal role for the somatic-to-germline transmission of environmental information capable of altering fetal development via changes in EV cargo. First, we showed that sperm miRNA content is impacted in an ongoing and persistent manner long after chronic stress exposure, corresponding with the timing of intergenerational transmission. We substantiated these data from our mouse model by showing in humans that sperm miRNA expression patterns were also associated with the dynamics of prior perceived stress experience and recovery. Next, we specifically focused on identifying the somatic involvement of the caput epididymis as the significant point in sperm post-spermatogenic maturation where (1) after spermatogenesis in the testes, signals from blood can interact with maturing sperm outside the blood-testes barrier, and (2) sperm gain critical required functions through signals from the epithelium, making this a specific epididymal region where sperm content may be most vulnerable to reprogramming processes. Modeling stress effects in an in vitro culture system, we demonstrated that the necessary component of the stress hormone signal, corticosterone, profoundly altered the composition of EVs secreted from EEC for delivery to maturing sperm, including changes in miRNA expression patterns and protein cargo, following a period of recovery from hormone exposure. Finally, we established causality of these EVs in signaling the paternal environment to germ cells for intergenerational transmission. Utilizing the artificial reproduction technique, intracytoplasmic sperm injection (ICSI), we showed that incubation of stress-naive sperm with EVs secreted by previously corticosterone-treated caput EEC was sufficient to significantly alter transcriptomic patterns in embryonic brain and placenta, suggestive of changes to neurodevelopment, and to recapitulate paternal stress germline transmission of a stress physiological phenotype.

Thus, we propose a model in mammals whereby environmental perturbations, such as stress, co-opt an existing signaling pathway along the reproductive tract to influence germ cell content. Our data suggest that this transmission is persistent, and programs phenotypes relevant to the originating environmental insult, effects that suggest passage of an adaptive advantage or vulnerability to future offspring, depending on their post-fertilization environment across their lifespan. Reprogramming of sperm miRNA populations by epididymal EVs are likely important in targeting important developmental pathways such that changes in an overall population of miRNA converge to produce important and sufficient paternal signals at fertilization and ultimately impact adult offspring phenotypes. For example, despite methodological distinctions between the stress paradigm and breeding timing in the current studies and those published previously from our lab over the last decade^10,30^ (differences in duration and severity of stress exposure and timing of breeding and intergenerational transmission following stress, and the removal of practice breeding experience), paternal transmission of offspring phenotypic changes in their HPA stress reactivity are consistent, suggesting that such environmental perturbations are relevant. Moreover, in the current study, sperm miRNA expression examined across time in both mice and humans suggests that ‘molecular aging’ of germ cells occur, similar to observations of oocytes and the brain^54,55^. Thus, perturbations such as stress may alter the trajectory of this maturational process, as suggested in age-by-disease models and studies showing the delayed effects of stress on the brain^56–58^, to impact germline transmission. We propose that somatic chromatin changes across time, suggested by caput epididymal histone PTM mass spectrometry data, may lie upstream of caput EEC EV content changes, ultimately impacting sperm miRNA and intergenerational transmission. Limitations of the current study are the lack of an in vivo manipulation of caput EEC EVs and maturing sperm, and a determination as to whether changes to caput EEC EV miRNA, proteins, and/or subtype composition (exosomes vs. microvesicles) are responsible for promoting intergenerational transmission. The answers to these questions, though beyond the scope of the current study, will be important as the field continues to define the molecular underpinnings of the intergenerational transmission of disease risk/resilience. Further, we propose that human sperm miRNA expression patterns may be used to detect prior experiences and exposures, offering the opportunity for future biomarker discovery in an easily collectable tissue, especially in at-risk populations that disproportionately experience stress or trauma.

## Methods

### Animals

Male C57BL/6J and female 129S1/SvImJ mice obtained from Jackson Laboratories were used to produce C57BL/6:129 F1 hybrids. F1 hybrids were used for all germline transmission studies. 6–8-week-old C57BL/6:129 F1 hybrid females (Jackson Laboratories, B6129SF1/J) were used for oocyte donation for ICSI studies. ICR female mice (Charles River, CD-1 IGS) were used as surrogates for embryo transfers for ICSI studies. All mice were housed in a 12:12 light:dark cycle with temperature 22 °C and relative humidity 42%. Food (Purina Rodent Chow; 28.1% protein, 59.8% carbohydrate, 12.1% fat) and water were provided ad libitum. All studies were performed according to experimental protocols approved by the University of Pennsylvania Institutional Animal Care and Use Committee, and all procedures were conducted in accordance with the NIH Guide for the Care and Use of Laboratory Animals.

### Chronic stress

Having solidified the sufficient duration of chronic stress required for intergenerational stress to occur (4 weeks vs. previous 6 weeks), we developed a model whereby we could investigate dynamic vs. long-lasting changes in tissues contributing to intergenerational transmission, using a time point post-stress that does not transmit a phenotype to offspring and a time point that did. At postnatal day 28 (PN28), males were weaned, pair-housed with a same-sex littermate, and randomly assigned to a control or stress group. Psychological stress occurred over 28 days (PN28-56). One stressor was administered each day and the order of stressors was randomized each week. Stressors include the following: 36 h constant light, 1 h exposure to predator odor (1:5000 2,4,5-trimethylthiazole (Acros Organics) or 1:2000 phenethylamine (Sigma)), 15 min restraint, novel object (marbles or glass vials) overnight, multiple cage changes, 100 dB white noise overnight, and saturated bedding overnight, as previously described^10,59^.

### Breeding scheme

Following completion of stress exposure (PN56), males were all left undisturbed for at least 1 week to remove the acute effects of stress. Males were then housed with virgin, stress-naive F1 hybrid females at either 9 or 20 weeks. To minimize male–female interactions that may impact maternal investment or care^60^, observation of a copulation plug within 1 h after lights on signaled the immediate removal of the female to her own cage containing a nestlet.

### Tissue collection

Males were rapidly decapitated under isoflurane anesthesia 24 h following copulation. The testes, caput and cauda epididymis were removed and flash frozen in liquid nitrogen. For E12.5 embryo collections, pregnant ICR surrogate dams were deeply anesthesized with isoflurane on E12.5, and each uterine horn was removed where conceptuses were harvested. Fetal brains, placentas and tails were flash frozen in liquid nitrogen and stored at −80 °C until processing. All dissections were completed between 11:00 and 15:00.

### Caudal sperm collection

Approximately 5 × 10^6^ sperm were collected from the caudal epididymis via a double swim-up assay. Briefly, the caudal epididymis was minced in 1% bovine serum albumin in 3 mL warmed PBS and allowed to sit at room temperature for 30 min. The 1% BSA including sperm and epididymal tissue were transferred to a conical tube and incubated in a water bath at 37 °C for 30 min. The top 2 mL of supernatant was transferred to a new conical tube and incubated at 37 °C for 10 min. The top 1.5 mL of this supernatant was transferred to a new tube and centrifuged for 5 min at 4000 rpm at 4 °C. The supernatant was removed and the pellet containing mature sperm was flash frozen and stored at −80 °C until processing. Sperm samples were not pooled for any experiments in these studies.

### HPA axis assessment

Plasma corticosterone was measured in response to an acute 15 min restraint stress in a 50 mL conical tube. Testing occurred 2–5 h after lights on. Tail blood was collected at onset and completion of restraint (0 and 15 min) and 15 and 115 min after the end of restraint (30 and 120 min). Samples were immediately mixed with 50 mM EDTA and centrifuged 10 min at 5000 rpm. Three microliters of plasma was collected and stored at −80 °C until analysis. Corticosterone levels were determined by ^125^I-corticosterone radioimmunoassay (MP Biomedical) according to manufacturer’s protocol. The *N* for each HPA axis assessment is as follows: Fig. 1b: 8 Control offspring and 8 Stress offspring (1 outlier); 1c: 8 Control offspring and 8 Stress offspring; 1d: 9 Control offspring and 7 Severe Stress offspring (1 outlier); 1e: 7 Control offspring (1 outlier) and 6 Severe Stress offspring; 3e: 5 EV^Veh^ offspring and 4 EV^Cort^ offspring.

### Cell culture and corticosterone treatment

Immortalized mouse distal caput epididymal epithelial (DC2) cells were purchased from Applied Biological Materials and cultured as previously described^61^. Briefly, cells were seeded in 75 cm^2^ Nunc EasYFlasks (Thermo Fisher) coated in collagen type 1, rat tail (Millipore). Cells were grown in Iscove’s modified Dulbecco’s medium (IMDM) supplemented with 10% exosome-free fetal bovine serum (Gibco) and 1% penicillin-streptomycin (Gibco). Fetal bovine serum was not charcoal-stripped and therefore contained base levels of steroids, including testosterone. At monolayer confluency, the media was replaced, and cells were either treated with 1:1000 vehicle (ethanol; resulting in 0.1% ethanol) or 1:1000 corticosterone in ethanol (Sigma; baseline concentration 144 nM, stress concentration 1.4 μM—resulting in 50 or 500 ng/mL of corticosterone in the culture media, respectively). Cells were treated every 24 h for 3 days for a total of three treatments. The media was replaced 24 and 96 h following the last treatment. Media and cells were collected at 24, 96, or 192 h following the last treatment and were not pooled. For cell collection, cells were trypsinized in 0.25% trypsin-EDTA (Gibco), centrifuged at 1500 rpm for 3 min, and frozen at −80 °C until further analysis.

### Extracellular vesicle (EV) isolation

EVs were isolated from conditioned media using differential ultracentrifugation^62^. Briefly, cellular debris was removed from the media by centrifugation at 200 × *g* for 10 min, 2000 × *g* for 10 min, and 10,000 × *g* for 30 min. EVs were pelleted by ultracentrifugation at 100,000 × *g* for 1 h using the Optima L-90K Ultracentrifuge and SW 32 Ti swinging bucket rotor (Beckman Coulter). The EV pellet was resuspended in PBS or TriZol reagent and frozen at −80 °C until further analysis. EVs were not pooled for any experiments in these studies.

### Protein extraction and western immunoblotting

EVs were processed for immunoblotting using established protocols. Samples were homogenized and resuspended in radioimmunoprecipitation assay (RIPA) buffer with protease inhibitor cocktail (Sigma), rotated for 2 h at 4 °C, and pelleted at 5000 × *g* for 10 min. Protein quantification was done using Bradford assay (BioRad). For immunoblotting, 20 μg of protein was loaded per lane for gel electrophoresis onto a NuPAGE 4–12% Bis-Tris gel (Life Technologies). After running, gels were cut and the same molecular weight sections for all samples were transferred together to enable multiple probing and to control for transfer conditions. After transfer of proteins to a nitrocellulose membrane (Life Technologies), membranes were blocked with Odyssey blocking buffer (Li-Cor) and probed with rabbit anti-CD63 (1:1000; Systems Biosciences EXOAB-CD63A-1), rabbit anti-Calnexin (1:1000; Abcam ab22595), and/or rabbit anti-Lamp1 (1:1000; Abcam ab24170), followed by incubation in IRDye800-conjugated donkey anti-rabbit secondary (1:20,000; Li-Cor).

### Nanoparticle tracking analysis

All samples were run on a NanoSight NS500 to determine the size distribution of EV particles at the Center for Nanotechnology in Drug Delivery at the University of North Carolina. All samples were diluted to a concentration between 1 × 10^8^ and 5 × 10^8^ particles/mL in filtered PBS. Five 40 s videos were taken of each sample to capture particles moving by way of Brownian motion. The nanosight software tracked the particles individually and, using the Stokes–Einstein equation, calculated the hydrodynamic diameters. The *N* for Fig. 2f, g: 4 Vehicle EV samples and 4 Stress Cort EV samples and samples were not pooled.

### IVIS spectrum imaging of labeled EVs

EVs isolated from cultured DC2 cells at day 11 (8 days post treatment) were labeled with XenoLight DiR Fluorescent Dye (PerkinElmer) per manufacturer’s instruction. Briefly, EV pellets were resuspended in 600 μL of cold PBS and incubated with 20 μL of 10 mM DiR dye for 5 min at RT. As a non-EV control, 600 μL of PBS alone was processed in parallel. The total volume was brought up to 38 mL with PBS and ultracentrifuged at 100,000 × *g* for 1 h. The dyed EV pellet was resuspended in PBS and 5 × 10^7^ particles were injected intravenously via the tail vein into naive adult F1 hybrid male mice. Twenty-four hours following injection, the mice were sacrificed and their tissues were collected for imaging using an IVIS Spectrum (PerkinElmer). The excitation filter was set at 745 nm and the emission filter was set at 800 nm. For quantification, total radiant efficiency was calculated using Living Image software, with the minimum set at 1 × 10^7^ and the maximum set at 1.45 × 10^7^. The N for IVIS experiments in Supplementary Fig. 5 is as follows: 6 injected Vehicle EVs, 6 injected Stress Cort EVs, where EVs were not pooled for any injection.

### Proteomics mass spectrometry

Individual EV samples were solubilized in 5% sodium deoxycholate after washing in phosphate-buffered saline. Proteins were washed, reduced, alkylated and trypsinolyzed on filter as previously described^63,64^. Tryptic peptides were separated on a nano-ACQUITY UPLC analytical column (BEH130 C18, 1.7 μm, 75 μm × 200 mm, Waters) over a 165-min linear acetonitrile gradient (3–40%) with 0.1% formic acid on a Waters nano-ACQUITY UPLC system and analyzed on a coupled Thermo Scientific Orbitrap Fusion Lumos Tribrid mass spectrometer as described^65^. Full scans were acquired at a resolution of 120,000, and precursors were selected for fragmentation by higher-energy collisional dissociation (normalized collision energy at 30%) for a maximum 3-s cycle. Tandem mass spectra were searched against a UniProt mouse reference proteome using Sequest HT algorithm^66^ and MS Amanda algorithm^67^ with a maximum precursor mass error tolerance of 10 ppm. Carbamidomethylation of cysteine and deamidation of asparagine and glutamine were treated as static and dynamic modifications, respectively. Resulting hits were validated at a maximum false discovery rate of 0.01 using a semi-supervised machine learning algorithm Percolator^68^. Label-free quantifications were performed using Minora, an aligned AMRT (Accurate Mass and Retention Time) cluster quantification algorithm (Thermo Scientific, 2017). Protein abundances were measured by comparing the MS1 peak volumes of peptide ions, whose identities were confirmed by MS2 sequencing as described above. The N for proteomics experiments is as follows: Fig. 2d left and Supplementary Figure 4: 5 Vehicle 4d EVs, 5 Stress Cort 4d EVs; Fig. 2d right, 2e: 6 Vehicle 11d EVs, 6 Stress Cort 11d EVs, where EVs were not pooled.

### Histone extraction, bottom-up nanoLC MS/MS

Samples were processed as previously described^69^. Briefly, whole caput epididymides were homogenized in nuclei isolation buffer (15 mM Tris-HCl pH 7.5, 60 mM KCl, 15 mM NaCl, 5 mM MgCl_2_, 1 mM CaCl_2_, 250 mM sucrose) with 1 mM DTT, 1% phosphatase inhibitor (Sigma), 1 pellet protease inhibitor (Roche), 10 mM sodium butyrate (Sigma), and 10% NP-40. Histones were acid extracted from nuclei by rotating overnight in 0.4 N H_2_SO_4_ at 4 °C and precipitated with 100% trichloroacetic acid overnight at 4 °C. Extracted histones were washed with acetone and quantified by Bradford reagent according to manufacturer’s protocol (Sigma). Approximately 20 μg histones were derivatized using propionic anhydride (Sigma) and digested with 1:10 trypsin (Promega). Samples were subsequently desalted by binding to C18 material from a solid phase extraction disk (Empore), washed with 0.5% acetic acid, and eluted in 75% acetonitrile and 5% acetic acid. Peptides were separated in EASY-nLC nanoHPLC (Thermo Scientific, Odense, Denmark) through a 75 μm ID × 17 cm Reprosil-Pur C_18_-AQ column (3 μm; Dr. Maisch GmbH, Germany) using a gradient of 0–35% solvent B (A = 0.1% formic acid; B = 95% acetonitrile, 0.1% formic acid) over 40 min and from 34 to 100% solvent B in 7 min at a flow-rate of 250 nL/min. LC was coupled with an Orbitrap Fusion mass spectrometer (Thermo Fisher Scientific, San Jose, CA, USA) with a spray voltage of 2.3 kV and capillary temperature of 275 °C. Full scan MS spectrum (*m*/*z* 300 − 1200) was acquired in the Orbitrap with a resolution of 60,000 (at 200 *m/z*) with an AGC target of 5 × 10e5. At Top Speed MS/MS option of 2 s, the most intense ions above a threshold of 2000 counts were selected for fragmentation with higher-energy collisional dissociation (HCD) with normalized collision energy of 29, an AGC target of 1 × 10e4 and a maximum injection time of 200 ms. MS/MS data were collected in centroid mode in the ion trap mass analyzer (normal scan rate). Only charge states 2–4 were included. The dynamic exclusion was set at 30 s. Where data-dependent acquisition^70^ was used to analyze the peptides, full scan MS (*m/z* 300–1100) was performed also in the Orbitrap with a higher resolution of 120,000 (at 200 *m*/*z*), AGC target set at the same 5 × 10e5. The difference in the MS/MS though also performed in the ion trap, was with sequential isolation windows of 50 *m/z* with an AGC target of 3 × 10e4, a CID collision energy of 35, and a maximum injection time of 50 ms. MS/MS data were collected in centroid mode. For both acquisition methods, peak area was extracted from raw files by using our in-house software EpiProfile^71^. The relative abundance of a given PTM was calculated by dividing its intensity by the sum of all modified and unmodified peptides sharing the same sequence. For isobaric peptides, the relative ratio of two isobaric forms was estimated by averaging the ratio for each fragment ion with different mass between the two species. Samples were not pooled.

### RNA isolation

Total RNA extraction from epididymal sperm and EV pellets were performed using the TRIzol reagent (Thermo Fisher) according to manufacturer’s protocol. Samples for all experiments represent individual samples and were not pooled.

### Incubation of caput epididymal sperm with DC2 EVs

Caput epididymal sperm were obtained by making incisions at both ends of the caput and through the tissue with a needle in modified Biggers, Whitten, and Whittingham media (BWW, composed of 91.5 mM NaCl, 4.6 mM KCl, 1.7 mM CaCl_2_.2H_2_O, 1.2 mM KH_2_PO_4_, 1.2 mM MgSO_4_.7H_2_O, 25 mM NaHCO_3_, 5.6 mM NaHCO_3_, 5.6 mM d-glucose, 0.27 mM sodium pyruvate, 55 mM sodium lactate, 5 U/ml penicillin/streptomycin, 20 mM HEPES buffer, 3 mg/ml BSA at pH 7.4) and left to ooze for 90 min at 37 °C, as previously described^48^. The whole volume (1 mL) was placed over a 3 mL 27% Percoll/BWW density gradient and spun at 400 × *g* for 15 min at RT. Spermatozoa in the pellet were washed again with BWW buffer and pelleted at 400 × *g* for 2 min. Using DC2 EVs extracted on the same day, individual caput epididymal sperm samples were split into two (not pooled) and co-incubated with 100 μL of either vehicle or corticosterone-treated DC2 EVs (~4 × 10^9^ total EVs) in a 96-well plate for 3 h at 37 °C with 5% CO_2_ with slight rotation, as previously described^72^. Following incubation, sperm were pelleted at 400 × *g* for 3 min and washed three times with warmed PBS. Clean sperm samples were then resuspended in the cryoprotective medium gCPA, containing 100 mM l-glutamine, and flash frozen in liquid nitrogen until intracytoplasmic sperm injection.

### Superovulation and oocyte collection

All the oocytes used for this experiment were collected from 6- to 8-week-old Bl6 x129 females (Jackson Laboratories). The donor females were super-ovulated using 5 IU of pregnant mare’s serum gonadotropin (PMSG) followed 48 h later by 5 IU of human chorionic gonadotropin (hCG). All the hormones were administrated via intraperitoneal injection IP. The oocytes were dissected from the ampulla of the oviduct 13–16 h post hCG and were placed in CZB-HEPES media supplemented with 3 mg/ml hyaluranidase to remove the cumulus cells. After 2–3 min incubation at room temperature, the oocytes were separated from the cumulus cells and immediately washed 3–4 times and cultured in KSOM media (Millipore) in a 37 °C, 5% CO_2_ incubator. The KSOM culture drops were covered with mineral oil (Millipore) to prevent evaporation.

### Intracytoplasmic sperm injection (ICSI) and embryo transfer

All the microinjections were performed at RT using a Narishige micromanipulator attached to a Nikon inverted microscope. To reduce the oocyte lysing rate, the CZB-HEPES media supplemented with 1% PVP was used for sperm injection and the 500–800 μL injection drop was covered with mineral oil to prevent evaporation; moreover, a glass-bottom dish was used to increase the resolution and contrast. The frozen sperm was washed once in CZB-HEPES media before it was placed in the injection drop on the microscope where the oocytes were added in groups of 10 to perform the microinjection. The sperm head was detached from tails by pining down the sperm to the bottom of the dish and applying some pressure right at the head/neck junction. The detached sperm heads were injected into the oocytes using an Eppendorf PiezoXper Microinjector. After injection the oocytes were placed back in the KSOM media in the 37 °C, 5% CO_2_ incubator where after 2–3 washes they were cultured 24–30 h until they were transferred into recipient females. Twenty-four to thirty hours post ICSI, all the embryos which successfully cleaved to the 2-cell stage were transferred into recipient females via oviduct transfer. Both, the left and the right oviduct were used for embryo transfers and the ICR recipient females were synchronized by using vasectomized males. CZB-HEPES media was used for embryo transfer.

### mRNA sequencing and analysis

Total RNA from E12.5 brains and placentas were quantified on a NanoDrop 2000 spectrophotometer (Thermo Scientific). Libraries for RNA sequencing were made using a TruSeq Stranded mRNA Sample Preparation Kit (Illumina) with 250 ng RNA according to manufacturer’s protocol. All library sizes and concentrations were confirmed on a TapeStation 4200 (Agilent) and Qubit 3.0 Fluorometer (Thermo Fisher). Individually barcoded libraries were pooled and and libraries for this study were sequenced on the same Illumina NextSeq 500 (75-bp single-end) flow cell, to control for batch effects. Fastq files containing an average of 50 million reads were processed for pseudoalignment and abundance quantification using Kallisto (version 0.43.1)^73^. The transcriptome was aligned to the EnsemblDB Mus musculus package (version 79). For mRNA sequencing, the *N* is as follows for each experiment: Fig. 3b–d and Supplementary Fig. 6: 6 ICSI EV^Veh^ E12.5 brains; 6 ICSI EV^Cort^ E12.5 brains; Supplementary Fig. 7: 6 ICSI EV^Veh^ E12.5 placentas; 6 ICSI EV^Cort^ E12.5 placentas where samples were not pooled.

### Gene set enrichment analysis (GSEA)

Gene set enrichment analysis^74^ (version 3.0, Broad Institute) was used to assess patterns of gene expression to determine greater-than-chance enrichment in biological pathways in a threshold-free manner (i.e., differential expression of genes was not considered). Total genes were run against the c5_BP gene set from the Molecular Signature Database (MsigDB v5.0, Broad Institiute) using 1000 gene_set permutations on E12.5 transcriptomic data and clustered to reduce redundancy using the ClusterMaker2 function in Cytoscape.

### Small RNA sequencing and analysis

Small RNA libraries were constructed using the NEBNext Small RNA Library Prep Set for Illumina (NEB) with 200 ng total RNA according to manufacturer’s protocol. All library sizes and concentrations were confirmed on a TapeStation 4200 (Agilent) and Qubit 3.0 Fluorometer (Thermo Fisher). Individually barcoded libraries were pooled and sequenced on an Illumina NextSeq 500 (75-bp single-end). Fastq files containing an average of 10 million reads per sample were aligned and quantified using miRDeep2 (version 2.0.0.8)^75^. For small RNA sequencing of sperm, the *N* is as follows: Fig. 1f: 7 Control 9-week samples, 6 Stress 9-week samples, 8 Control 20-week, 8 Stress 20-week (1 outlier). For sequencing of DC2 EVs, the *N* is as follows: Fig. 2b, c, Supplementary Fig. 3: 4 days—3 Vehicle EVs, 4 Baseline Cort EVs, and 4 Stress Cort EVs; 7 days—4 Vehicle EVs, 4 Baseline Cort EVs, and 4 Stress Cort EVs; 11 days—4 Vehicle EVs, 4 Baseline Cort EVs, and 3 Stress Cort EVs. Samples were not pooled. For each study, samples from all treatment groups were represented in each of two flow cells so that each treatment group was equally represented per sequencing run to control for batch effects.

### Taqman miRNA assay

To confirm observed changes in the sperm and DC2 EV RNA-sequencing data, miRNA displaying effects of age (miR-741-3p and miR-881-3p), stress (miR-34c-5p and miR-9-3p), or corticosterone (miR-22-3p and miR-34c-5p) treatment were assayed by reverse-transcription quantitative real-time PCR (RT-qPCR) in conjunction with TaqMan miRNA Assays (Applied Biosystems) according to the manufacturer’s protocol. Selected miRNA had high expression levels (ranging from 70 to 40,000 normalized reads) and displayed robust effects by age or treatment (log_2_ fold change > |0.40|) by RNA sequencing. Briefly, 350 ng of total RNA was reverse transcribed to cDNA using the Taqman MicroRNA Reverse Transcription Kit (Applied Biosystems) and a pool of assay-specific reverse transcriptase primers. Prior to the real-time PCR reaction, the RT product was amplified using the Taqman PreAmp Master Mix (Applied Biosystems). PCR reactions, including two endogenous controls (U6 snRNA and snoRNA-202), were run using TaqMan Universal Master Mix II, no UNG (Applied Biosystems) on a QuantStudio 5 Real-Time PCR System. All Taqman reactions were run in triplicate in 384-well plates, where samples were distributed across two plates so that each treatment group was equally represented per run to control for batch effects. Ct values were calculated using the instrument’s onboard software. The mean Ct values for the two endogenous controls were subtracted from corresponding Ct values of the miRNA of interest. Resulting ΔCt values were used to calculate expression (relative to Control 9-week) using the ΔΔCt method. To correlate Taqman qPCR with RNA-sequencing results, the log_2_ values of the qRT-PCR fold change (relative to 9-week control or vehicle EVs) were plotted against the log_2_ RNA-sequencing read counts for selected miRNA and examined using linear regression analysis. For the Taqman miRNA assays in sperm, the *N* was as follows: Supplementary Fig. 2b: 7 Control 9-week samples, 8 Control 20-week samples, 6 Stress 9-week samples, and 7 Stress 20-week samples, where sperm samples were not pooled; and 4 Vehicle EV samples and 3–4 Corticosterone EV samples, where EV samples were not pooled.

### Bioinformatics analyses

All analyses were performed using R version 3.3.3 and Bioconductor version 3.4.

### Rank–rank hypergeometric overlap (RRHO)

The R package RRHO2 was used to evaluate the degree and significance of overlap in threshold-free differential expression data (nominal *p*-values for all miRNA from Control vs Stress comparisons using DEseq analysis) between in vivo sperm and in vitro EV miRNA datasets^46,47^. This updated RRHO pipeline improves the visualization of overlap heatmaps, where pixels are plotted in one of four quadrants in order to determine the concordance and directional overlap between datasets, compared with prior uses of the original RRHO analysis where pixels were continuously plotted without distinctive areas for each categorization (hence, one heatmap with no separation). Heatmaps generated using RRHO2 have top right (both increasing) and bottom-left (both decreasing) quadrants, representing the concordant miRNA changes, while the top left and bottom right represent discordant overlap (opposite directional overlap between datasets). For each comparison, one-sided enrichment tests were used on −log_10_(nominal *p*-values) with the default step size for each quadrant, and corrected Benjamini–Yekutieli *p*-values were calculated. To ensure RRHO-identified significant overlap between sperm and DC2 EV miRNA were detected above chance, EV miRNA samples were randomly assigned to groups and the same analysis was rerun on nominal *p*-values of all detected miRNA, where randomization was used on Vehicle and Stress Cort EV miRNA samples within time at 8 days post treatment, on Vehicle, Baseline Cort, and Stress Cort EV miRNA samples within time at 8 days post treatment, and on Vehicle and Stress Cort EV miRNA samples across time such that 1 Vehicle and 1 Stress Cort sample were randomly selected from 1, 4, and 8 days post treatment. The number of concordant EV miRNA for each analysis was quantified and used to calculate the percentage of concordant miRNA over total identified miRNA

### Differential expression analysis

The R package DESeq was used to perform pairwise differential expression analyses on RNA-sequencing datasets using the negative binomial distribution^76^. For E12.5 brain mRNA sequencing, count data were filtered for at least 10 counts per gene across all groups, normalized, and dispersions were estimated per condition with a maximum sharing mode. Small RNA-sequencing data from mouse sperm and DC2 cell EVs were filtered for >2 counts in at least three samples across all groups, normalized, and dispersions were estimated per condition using empirical values. Significance for all differential expression was set at a corrected *p*-value < 0.05. Heatmaps were generated using the R package gplots heatmap.2 function. All heatmaps are plotted as average Z scores per treatment group.

### Random forests

The R package randomForest^45^ was used to analyze histone mass spectrometry ratio data with the parameters ntree = 1000 and mtry = √*p* for classification analysis, based on calculation of *p* where *p* = total number of histone modifications identified. Importance values were calculated and scaled by standard deviation for permutation-based measures. This approach ranks each histone modification by the percent decrease (MDA) to the model’s accuracy that occurs if the histone mark is removed, allowing for the identification of a histone code that discriminates between treatment groups. To estimate the minimal number of histone modifications required for prediction, ten-fold cross-validation using the ‘rfcv’ command was implemented through the randomForest package.

### Statistics

Investigators blinded to animal treatment groups conducted all experiments and analyses. Samples for all experiments represent individual samples and were not pooled. For the following, statistical comparison was conducted using Prism 7.0 (Graphpad). Corticosterone levels were analyzed by two-way ANOVA with time as a repeated measure. Corticosterone AUC, litter characteristics, and gene expression data were analyzed by two-way ANOVAs. Outliers for HPA axis assessment were excluded at all time points and determined by data greater than two standard deviations away from the group mean or corticosterone levels >150 ng/mL at the 120 min time point, indicating no stress recovery and/or within littermate fighting that were noted at time of experiment (for 9-week HPA, 1 Stress offspring and 1 Severe Stress offspring, for 20-week HPA, 1 Control offspring). Taqman miRNA qRT-PCR data were analyzed using two-way ANOVA for sperm and unpaired two-tailed Student’s *t*-test for EVs. Immunoblotting data, nanosight, and IVIS radiant efficiency were analyzed using unpaired two-tailed Student’s *t*-tests. When appropriate, Bonferroni’s post hoc test was used to explore main effects followed by multiple comparisons adjustment (denoted adjusted *p*). Significance was set at *p* < 0.05.

### Recruitment of human subjects

A cohort of 18 healthy males had been recruited from the University of Pennsylvania student body to establish normative sperm molecular signatures as a benchmark for comparison to later clinical populations. The study was approved by the Perelman School of Medicine at the University of Pennsylvania Institutional Review Board, and all participants provided written informed consent. Subjects between the ages of 18 and 25 were screened for history of major medical illnesses, mental health diagnoses, and substance abuse. The participants were English-speaking and gave written informed consent for participation in this study, which was approved by the Perelman School of Medicine at the University of Pennsylvania Institutional Review Board. Key exclusion criteria included (1) history of major medical illnesses, including liver diseases, suspected or known malignancy, pulmonary disorders, clotting or bleeding disorders, heart disease, diabetes, history of stroke or other medical conditions that the physician investigator deemed as contraindicated for the patient to be in the study, as determined by participant self-report; (2) regular or recreational use of psychotropic medication (antidepressants, antipsychotics, or anxiolytics), as per self-report, and recent (within previous year) psychiatric diagnosis and treatment for Axis I disorders including major depression, bipolar disorder, generalized anxiety disorder, post-traumatic stress disorder, and panic disorder, as determined by MINI International Neuropsychiatric Interview^77^; (3) lifetime history of schizophrenia or other psychotic disorder, as per MINI International Neuropsychiatric Interview; (4) lifetime substance addiction disorder, excepting nicotine, as per MINI International Neuropsychiatric Interview; (5) substance abuse disorders within the previous 2 years, excepting nicotine, as per MINI International Neuropsychiatric Interview; (6) use of any tobacco products, determined by urine cotinine level; (7) positive drug screen for any substance, determined by urine drug screen at screening.

### Study procedures for human subjects

The study involved a total of seven visits. The first visit was a screening visit to determine participant eligibility. The following six visits were sperm collection visits. During the screening visit, subjects underwent an in-office assessment including a urine toxicology screen, urine cotinine screen, and clinical assessments, including the Adverse Childhood Experiences (ACE) questionnaire^43^ and the MINI International Neuropsychiatric Interview. Subsequent visits (2–7) took place once a month for 6 months. At these visits, subjects submitted a semen sample, collected at home within the previous hour, to experienced andrologists at Penn Fertility Care clinic for processing and sample cryopreservation. Participants were asked to abstain from ejaculation for 48 h prior to semen collection. Within the same day, participants also completed a series of questionnaires to assess stress and anxiety experienced over the previous month, including the Perceived Stress Scale (PSS)^41^ and the Spielberger State-Trait Anxiety Inventory^42^ (STAI). One participant did not return for their final donation, therefore only timepoints 1–5 were available for subject 11.

### Assessing the impact of stress dynamic on human sperm miRNA

Procedures for the isolation of small RNA from mature sperm were adapted from a previous study^78^. Briefly, cryopreserved sperm samples were thawed, suspended in PureSperm Buffer (Nidacon), then mature sperm were enriched by centrifugation (300 × *g*, 15 min) through a 50% PureSperm density gradient (Nidacon). Sperm were then lysed in TRIzol-LS (Thermo Fisher) reagent, supplemented with 0.2 M β-mercaptoethanol and 100 mg of nuclease-free stainless-steel beads, by homogenization on a Disruptor Genie (Scientific Industries) at 3000 rpm for 5 min. RNA, enriched for small RNA, was isolated using Qiagen’s miRNeasy Mini kit according to manufacturer’s instructions. RNA concentration and quality were assessed using Agilent’s small RNA chipsrun on a Bioanalyzer 2100 (Agilent Technologies). Three subjects (4, 6, and 15) were excluded from further analysis due to consistently low RNA yield and quality across donated samples. PSS scores reported by the remaining subjects over the 6-month study period were assessed to identify subjects with a stress experience dynamic that best mimicked our mouse model. Four subjects (1, 7, 12, and 18) that experienced elevated perceived stress early in the study, followed by a period of recovery (recovering-stress dynamic) (defined as a change in PSS score >10 between the max PSS score early in the study (month 1 or 2) and minimum PSS score late in the study (month 5 or 6)). Four individuals (2, 5, 11, and 14) with minimal variation in reported PSS scores over the study (standard deviation from the mean PSS score <2) were selected to make up a comparison stable-stress dynamic group. The small RNA content of samples from these two groups of subjects was analyzed by small RNA sequencing. Libraries were constructed using the TruSeq small RNA Library Prep Kit (Illumina) with 10 ng of small RNA according to the manufacturer’s protocol. Post-PCR cleanup and size selection for products >100 bp was performed using AMPure XP bead purification. Library size distribution and quantification was performed using on a TapeStation 4200 (Agilent) using their High Sensitivity D1000 screentape. Individually barcoded libraries were pooled to achieve ~10 million reads per sample and sequenced on an Illumina NextSeq 550 (36-bp single-end).

### Bioinformatic analysis of human sperm miRNA

Because sequence reads were 36 bp, which is greater than the average size of miRNA (22 bp), reads were trimmed at the 3′ end to remove any trailing adaptor sequence using the Trimmomatic tool^79^. After read trimming, reads shorter than 15 bp were discarded before downstream analyses. One sample was excluded at this stage for quality concerns (sample from subject 5 at collection 4 had <1 million remaining reads). Trimmed reads were then aligned to the human reference genome build GRCh38 using the Bowtie short read aligner^80^. Reads were aligned allowing for 2 mismatches and a seed length of 15. The expression of each known miRNA from miRbase v22^81^ were computed using HTSeq python framework^82^. Normalized expression counts for miRNA detected in >50% of samples (341 miRNA) were further analyzed by implementing a linear mixed effects model using the R package ‘lme4’, accounting for the repeated measures structure of the data by treating subject as a ‘random’ effect^83^. Using this modeling strategy, we tested the association between stress-experience dynamic group assignment and principal components identified in an unbiased dimensional reduction analysis (PCA analysis) of the expression of total miRNA across samples.

### Reporting summary

Further information on research design is available in the Nature Research Reporting Summary linked to this article.

## Supplementary information

Supplementary Information

Description of Additional Supplementary Files

Supplementary Data 1

Supplementary Data 2

Supplementary Data 3

Supplementary Data 4

Supplementary Data 5

Supplementary Data 6

Supplementary Data 7

Supplementary Data 8

Supplementary Data 9

Reporting Summary

## Data Availability

Raw and processed sequencing data are available from the Gene Expression Omnibus (GEO) database under accession code GSE145051. Raw data for histone post-translational modification and protein mass spectrometry experiments are provided as Supplementary Data 4 and 6, respectively. All other relevant data supporting the key findings of this study are available within the article and its Supplementary Information files or from the corresponding author upon reasonable request. Source data underlying Supplementary Fig. 3 are provided as a source data file. The Molecular Signature Database (MsigDB v5.0) is available through the Broad Institute. A reporting summary for this Article is available as a Supplementary Information file.

## Source data

Source Data

## Code availability

All data analyses used were from published software. Requests can be sent to the corresponding author.
