# Supplementary material for: Reproductive tract extracellular vesicles are sufficient to transmit intergenerational stress and program neurodevelopment
Source: Nat Commun. 2020 Mar 20;11:1499. doi: 10.1038/s41467-020-15305-w (PMC7083921; doi:10.1038/s41467-020-15305-w)
Supplement: Supplementary file 1 — Supplementary Information [file 41467_2020_15305_MOESM1_ESM.pdf]

Supplementary Information for

Reproductive tract extracellular vesicles are sufficient to transmit intergenerational stress and program neurodevelopment

Jennifer C Chan[1], Christopher P Morgan[2], N Adrian Leu[1], Amol Shetty[3], Yasmine M Cisse[2], Bridget M Nugent[2], Kathleen E Morrison[2], Eldin Jašarević[2], Weiliang Huang[4], Nickole Kanyuch[2], Ali B Rodgers[1], Natarajan V Bhanu[5], Dara S Berger[6], Benjamin A Garcia[5], Seth Ament[3], Maureen Kane[4], C Neill Epperson[7], Tracy L Bale[2]

correspondence to: tbale@som.umaryland.edu

This file includes:

Supplementary Figures and Legends: 1-9

Other Supplementary Materials for this manuscript includes the following:

Supplementary Data 1: Results table from DEseq analysis of sperm miRNA

Supplementary Data 2: Statistics for qRT-PCR validation of sperm and EV RNA-sequencing

Supplementary Data 3: Baseline demographics and assessments including ACE questionnaire and STAI inventory for all subjects in human cohort

Supplementary Data 4: Data for raw caput epididymal histone mass spectrometry data

Supplementary Data 5: Results table from DEseq analysis of DC2 EV miRNA

Supplementary Data 6: Data for raw DC2 EV protein mass spectrometry data

Supplementary Data 7: GO terms for Figure 3e, E12.5 offspring brains from ICSI of sperm incubated with EVs collected from DC2 cells following treatment

Supplementary Data 8: Complete result Data for GO terms in E12.5 offspring brains from ICSI of sperm incubated with EVs collected from DC2 cells following treatment

Supplementary Data 9: Complete result Data for GO terms in E12.5 offspring placenta from ICSI of sperm incubated with EVs collected from DC2 cells following treatment

Supplementary Figure 1

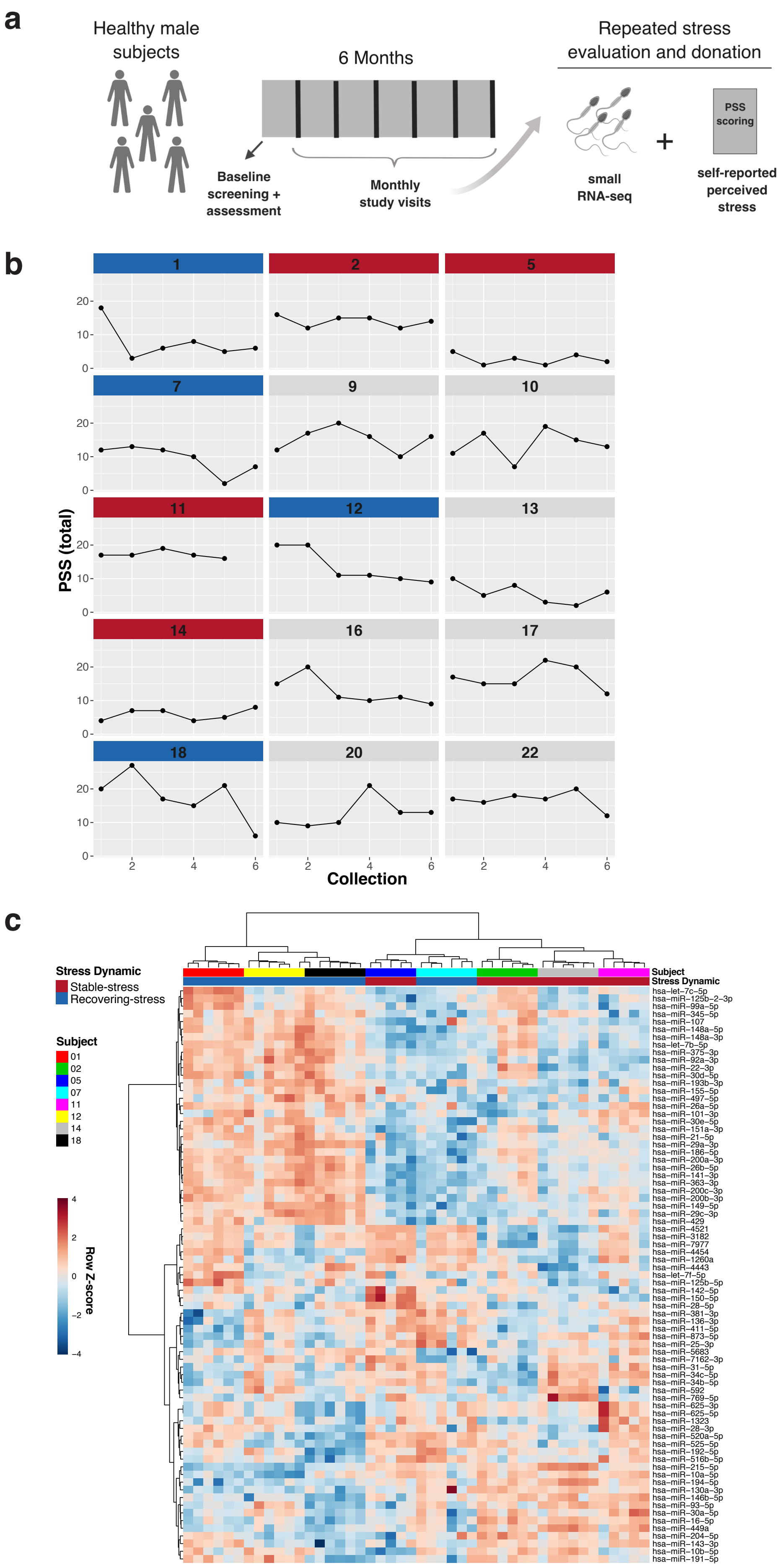

Supplementary Figure 1. Between- and within-subject variation in human sperm miRNA expression patterns over time. (**a**) To translationally probe our mouse model and examine the impact of prior stress experience and recovery on human sperm miRNA patterns, recruited healthy male subjects (N=15) completed monthly psychological inventories, including the Perceived Stress Scale (PSS), and donated sperm samples over 6 months. (**b**) From self-reported PSS scores, we identified two phenotypic groups of subjects from which we could probe our mouse model findings: 1) One group of subjects, which best mimicked our mouse model, reported elevated perceived stress followed by an extended period of recovery (recovering-stress), defined as a drop in PSS score ≥ 10 over the 6 months (blue bars, N=4), and 2) A comparison group with minimal variation in PSS score, regardless of the intensity of that stress (stable-stress) over 6 months (red bars, N=4). (**c**) Connections between subject sperm miRNA expression patterns are visualized in a heatmap of the 75 miRNA with the greatest between-subject variation (ranked by one-way ANOVA). Hierarchical clustering identified a single cluster that includes all subject samples (6 from each) from 3 of 4 subjects with a stable-stress dynamic, while excluding all others, and a second cluster was comprised of all samples from recovering-stress subjects that also included one of the stable-stress subjects (N=24 samples from stable-stress and N=22 samples from recovering-stress groups).

Supplementary Figure 2

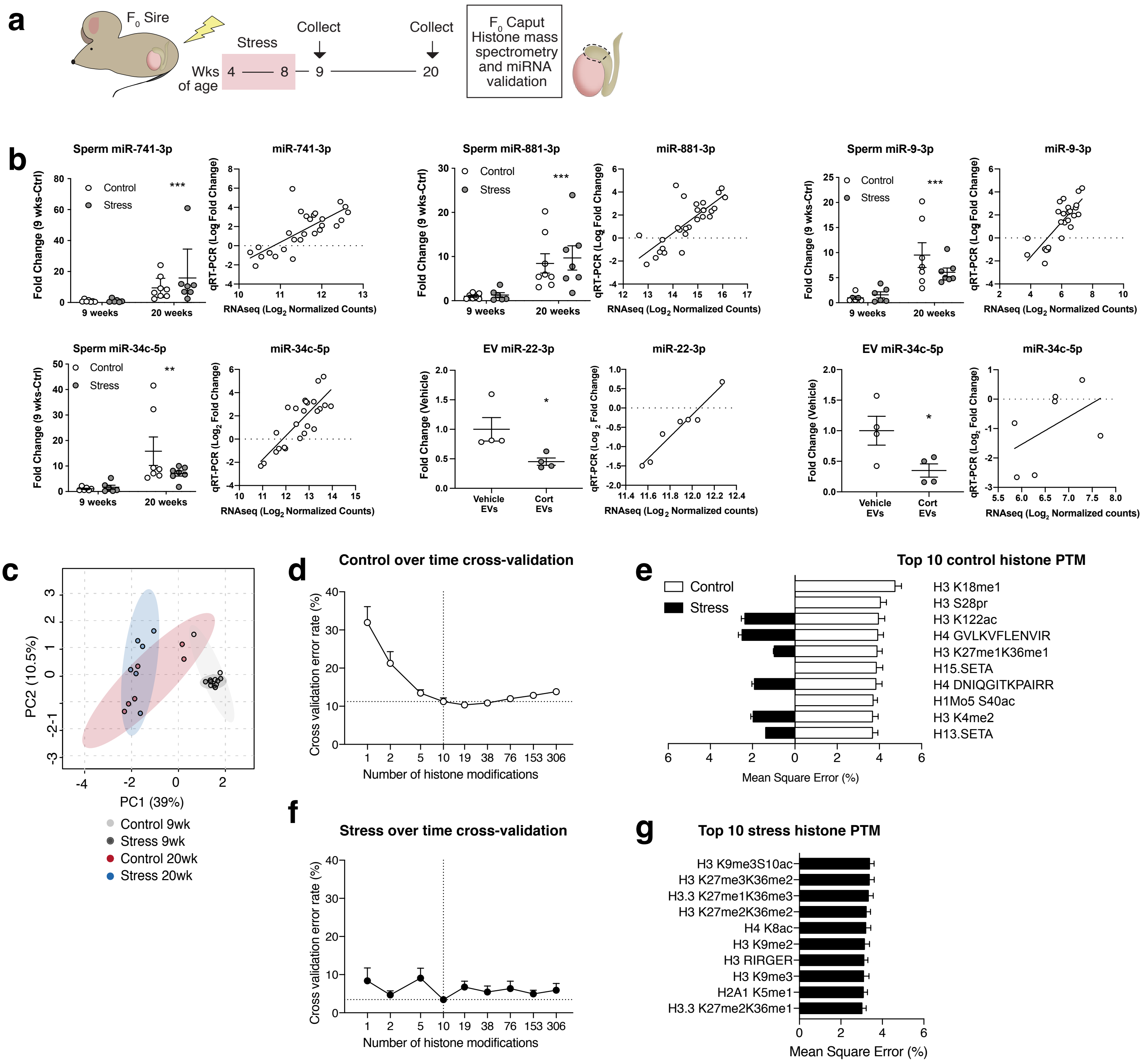

Supplementary Figure 2. Stress recovery impacts histone modifications during epididymal maturation. (**a**) Male mice experienced chronic stress from age 4-8 weeks and caput epididymal tissues were collected at 9- or 20-weeks. Histone post-translational modification (PTM) mass spectrometry was used to examine caput epididymal histone PTM profiles following stress and recovery. (**b**) Validation of age- (miR-741-3p and miR-881-3p) and stress- (miR-9-3p and miR-34c-5p) related differences in sperm miRNA and corticosterone-related differences in DC2 EVs (miR-22-3p and miR-34c-5p) by quantitative RT-PCR. See methods for Ns. Full statistics are provided in Supplementary Table 2. Error bars represent mean ± SEM. (**c**) Unbiased principle components analysis of caput epididymal tissues at 9- and 20-weeks based on all detected histone PTMs, showing divergence following recovery but not immediately following stress (ellipses are 95% confidence intervals). (**d**) Histone PTM ratios were analyzed by Random Forests, identifying the top ten histone PTMs that best described epididymal tissue maturation from 9-to-20-weeks (i.e. identify the top ten histone PTMs that contribute to accuracy of a control epididymal maturation model). Cross-validation over ten iterations, where 10 histone PTMs were the minimal necessary features for greatest model accuracy. **(e)** The top 10 histone PTMs plotted (white bars), ranked by importance to normal epididymal maturation. The black bars are the corresponding importance of each histone PTM from a stress epididymal maturation model, showing that these histone PTMs contribute less or no accuracy in epididymal tissues recovering from stress. Importance is % increase in mean-squared error of epididymal maturation model when PTM values were randomly permuted. **(f)** Cross-validation of the stress epididymal maturation model, where 10 histone PTMs were the minimal necessary features for

greatest model accuracy. **(g)** The top 10 histone PTMs, ranked by importance to stress recovery in epididymal tissue, demonstrating the top histone PTMs changing during stress recovery are largely distinct from those changing in control epididymal tissues, suggesting stress recovery programs a new allostatic set point in the caput epididymis. **(d,f)** Error bars represent mean ± SEM, N=10 iterations. **(e,g)** Error bars represent SD used to scale importance values, with Random Forests analysis performed on N=6 epididymal tissues/treatment/age.

Supplementary Figure 3

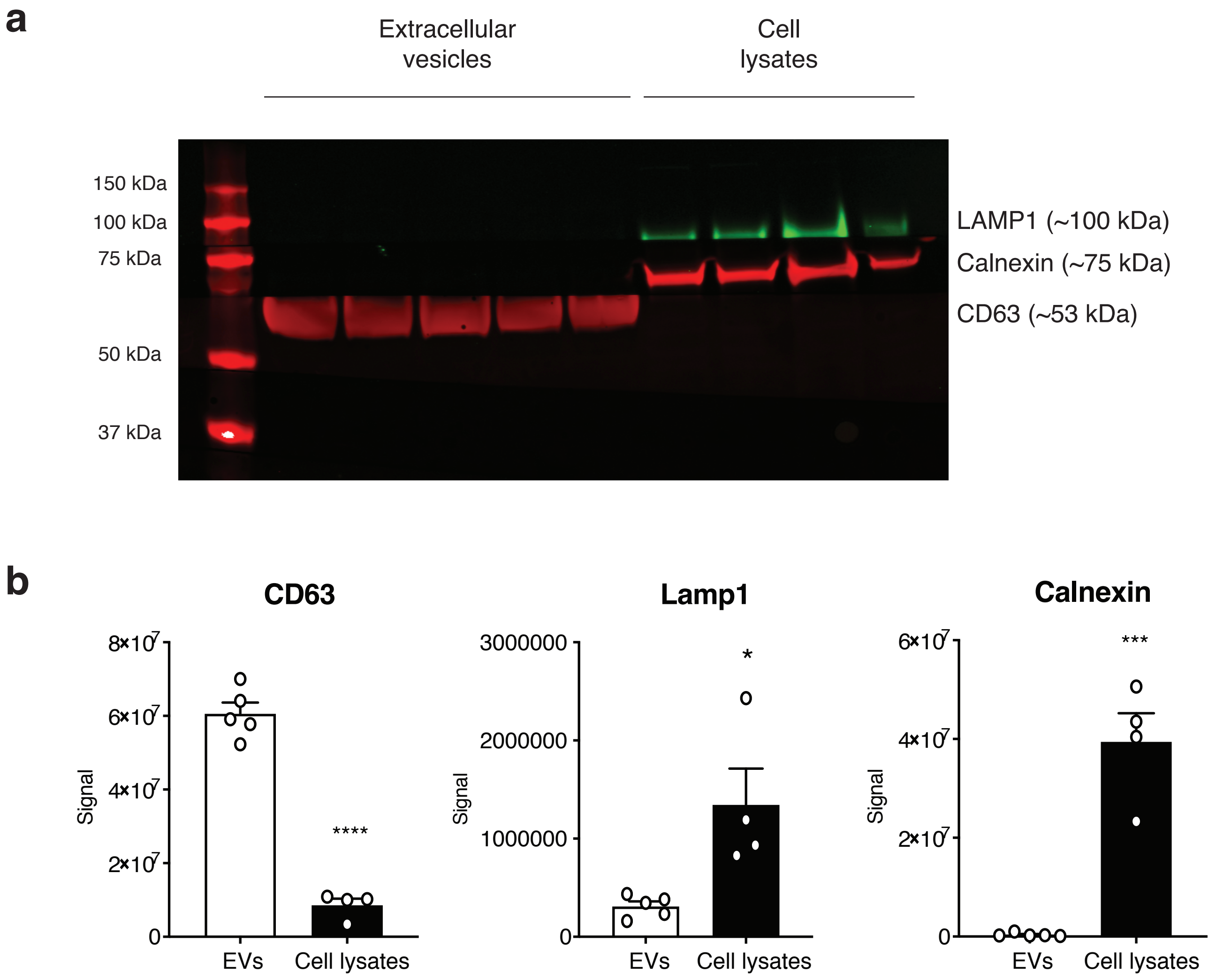

Supplementary Figure 3. Validation of extracellular vesicles (EV) isolated from culture media of DC2 caput epididymal epithelial cells. (**a**) Representative western blot and (**b**) quantification of CD63, a known EV-enriched tetraspanin; Calnexin, an endoplasmic reticulum-associated protein; and Lamp1, a lysosome-associated protein. CD63 (unpaired two-tailed Student's t-test, $t(7)=13.96$, ****$p=2.2902\times10^{-6}$) is typically found on EV membranes, while Calnexin (unpaired two-tailed Student's t-test, $t(7)=7.678$, ***$p=0.0001$) and Lamp1 (unpaired two-tailed Student's t-test, $t(7)=3.138$, *$p=0.0164$) are typically found from cell lysates, suggesting minimal cellular contamination in isolated EV populations. N=5 cellular lysate samples and 4 EV samples. Error bars represent mean ± SEM, with individual data points overlaid. Source data are provided as a Source Data file.

Supplementary Figure 4

a

Overlap of sperm miRNA and EV miRNA (Vehicle vs. Baseline Cort)

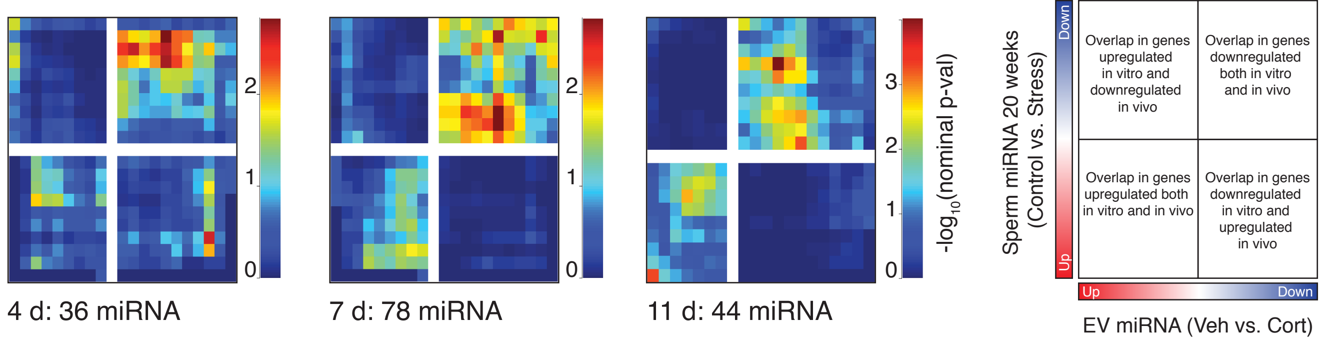

b

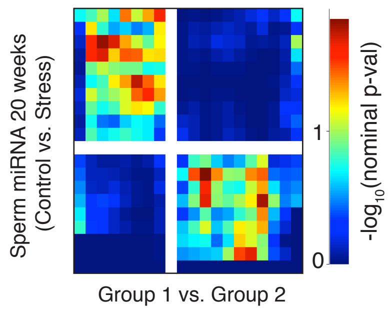

Number concordant miRNA: 3 (3/369 = 0.8%)

Group 1	Veh d11	Stress Cort d11	Veh d11	Stress Cort d11
Group 2	Stress Cort d11	Veh d11	Stress Cort d11	

c

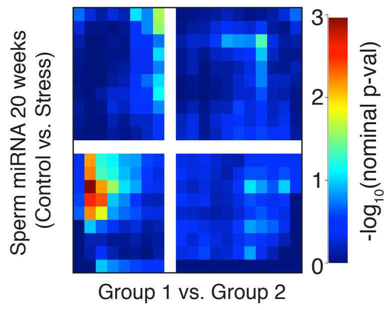

Number concordant miRNA: 27 (27/369 = 7.3%)

Group 1	Veh d11	Low Cort d11	Veh d11	Stress Cort d11
Group 2	Low Cort d11	Veh d11	Stress Cort d11	

d

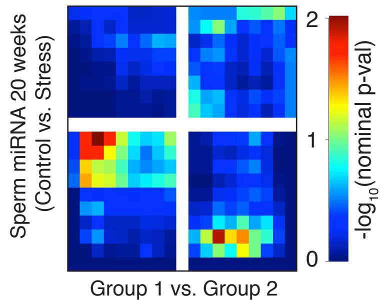

Number concordant miRNA: 30 (30/340 = 8.8%)

Group 1	Veh d4	Veh d7	Veh d11
Group 2	Stress Cort d4	Stress Cort d7	Stress Cort d11

Supplementary Figure 4. Quantification of miRNA overlap between paternal stress sperm and baseline corticosterone treatment of DC2 EVs. (**a**) To quantify concordant miRNA overlap between our mouse model and cell culture, RRHO analysis was used. Following small RNA sequencing, the differential expression profiles of sperm 12-weeks post-stress were plotted against the differential expression miRNA profiles of secreted EVs 1, 4, or 8 days following baseline corticosterone treatment (left, middle, and right respectively). Overlap data are plotted as sperm miRNA ratios increasing down the y-axis and EV miRNA ratios increasing left along the x-axis, with each pixel representing the -$\log_{10}$(nominal p-value) of overlapping miRNA via the hypergeometric distribution and the color coding according to degree of significance (as shown). Each RRHO heatmap is divided into four quadrants, where the bottom left and upper right squares represent concordant miRNA changes in both models as quantified below each heatmap. (**b-d**) To ensure RRHO-identified significant overlap between sperm and DC2 EV miRNA were detected above chance, EV miRNA samples were randomly assigned to groups and the same analysis was rerun on nominal p-values of all detected miRNA, where randomization occurred (**b**) using Vehicle and Stress Cort EV miRNA samples within time at 8 days post-treatment, (**c**) using Vehicle, Baseline Cort, and Stress Cort EV miRNA samples within time at 8 days post-treatment, and (**d**) using Vehicle and Stress Cort EV miRNA samples across time such that 1 Vehicle and 1 Stress Cort sample were randomly selected from 1, 4 and 8 days post-treatment. The samples included in each group for this randomized comparison are depicted under each heatmap. The number of concordant EV miRNA for each analysis was quantified and used to calculate the percentage of concordant miRNA over total identified miRNA (where total miRNA are

miRNA present in every sample in that comparison), showing that the percentage for each of these randomized analyses (0.8-8.8%) were below that identified at 8-days post-treatment in the Stress Cort comparison with sperm (31.4%). (N=3-4 EVs/treatment/time, max $-\log_{10}$(p-value) = 4).

Supplementary Figure 5

a

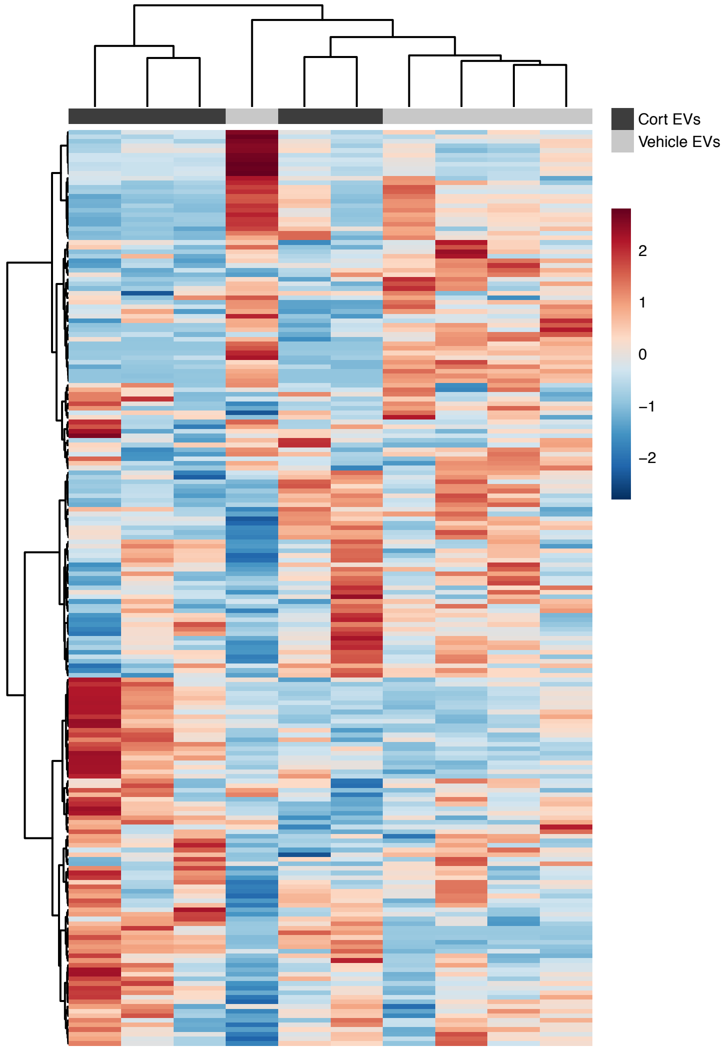

Supplementary Figure 5. DC2 EV protein cargo 1-d following corticosterone treatment. (**a**) Heatmap and hierarchical clustering of all detected proteins from proteomics mass spectrometry of DC2 EVs collected acutely 1 day following corticosterone treatment (N=5 EVs/treatment), showing the effects of corticosterone treatment on EV protein content is greater at 8 days post-treatment (as shown in Figs. 2d, e).

Supplementary Figure 6

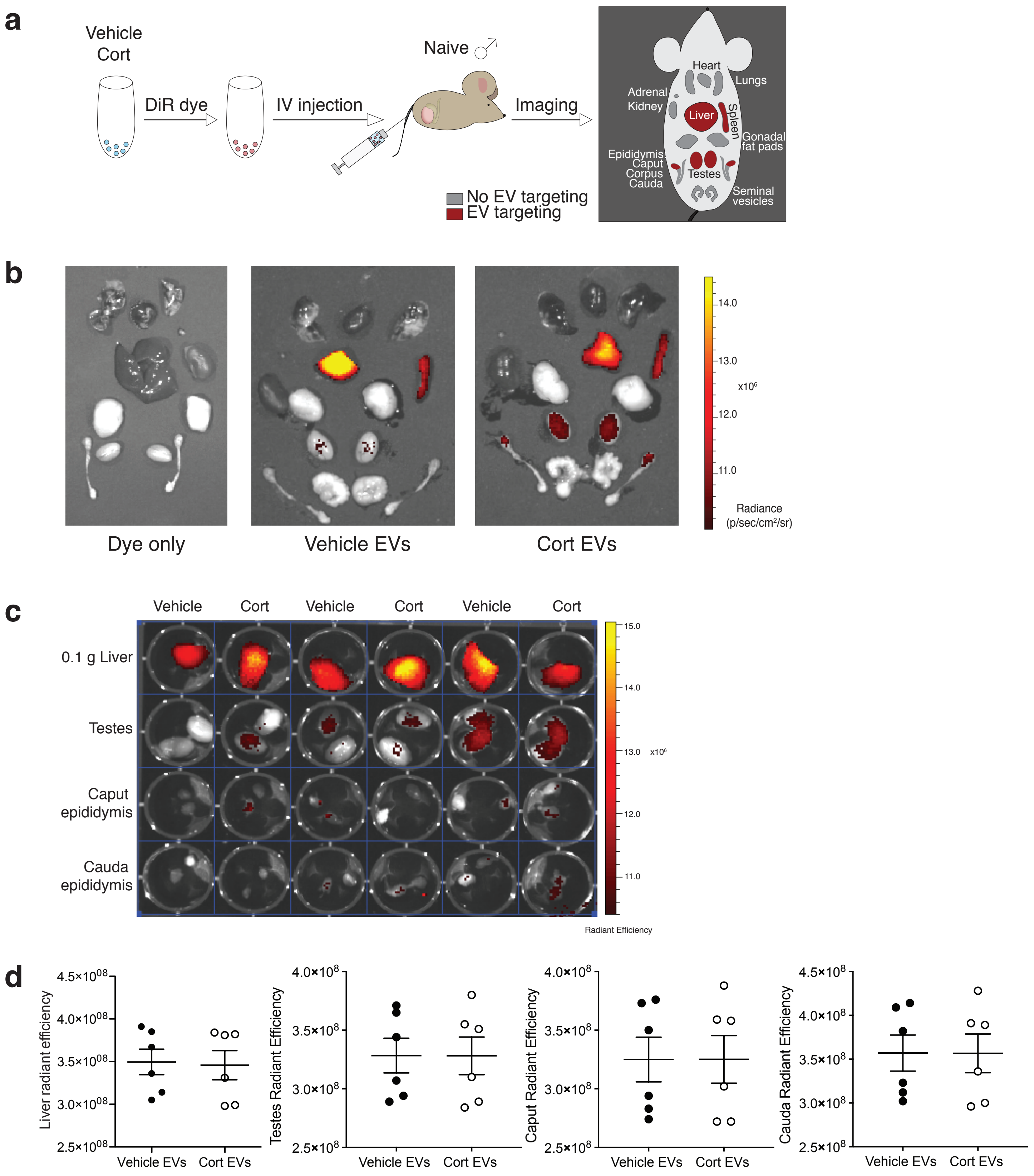

Supplementary Figure 6. Imaging and quantification of tissues from naive male mice injected i.v. with DiR-labeled EVs secreted from DC2 caput epididymal epithelial cells. (**a**) To ensure EVs treated with stress corticosterone levels maintained tissue targeting specificity *in vivo*, we fluorescently labeled vehicle- and stress corticosterone-treated DC2 EVs with the near-infrared lipophilic DiR dye and injected 5 x 10^7 EVs intravenously into naïve male mice. 24-hrs post-injection, tissues were removed and imaged to evaluate the bio-distribution of DC2 caput EEC EV targeting. (**b**) Representative images of the biodistribution of DC2 EVs following the i.v. infusion of DiR dye-labeled EVs collected 8 days after vehicle or stress corticosterone treatment, demonstrating that EVs retained their tissue targeting selectivity. (**c**) Liver, testes, caput and cauda epididymal tissue from mice injected with DC2 EVs collected 8-days after either vehicle or corticosterone treatment. (**d**) There were no statistically significant differences in total radiant efficiency of liver (unpaired two-tailed Student's t-test, $t(10) = 0.1691$, $p = 0.8691$), testes (unpaired two-tailed Student's t-test, $t(10) = 0.007625$, $p = 0.9941$), caput epididymis (unpaired two-tailed Student's t-test, $t(10) = 0.005991$, $p = 0.9953$), or cauda epididymis (unpaired two-tailed Student's t-test, $t(10) = 0.01103$, $p = 0.9914$), showing no changes to EV tissue targeting selectivity by DC2 treatment. N=6 mice/EV treatment. Error bars represent mean ± SEM.

Supplementary Figure 7

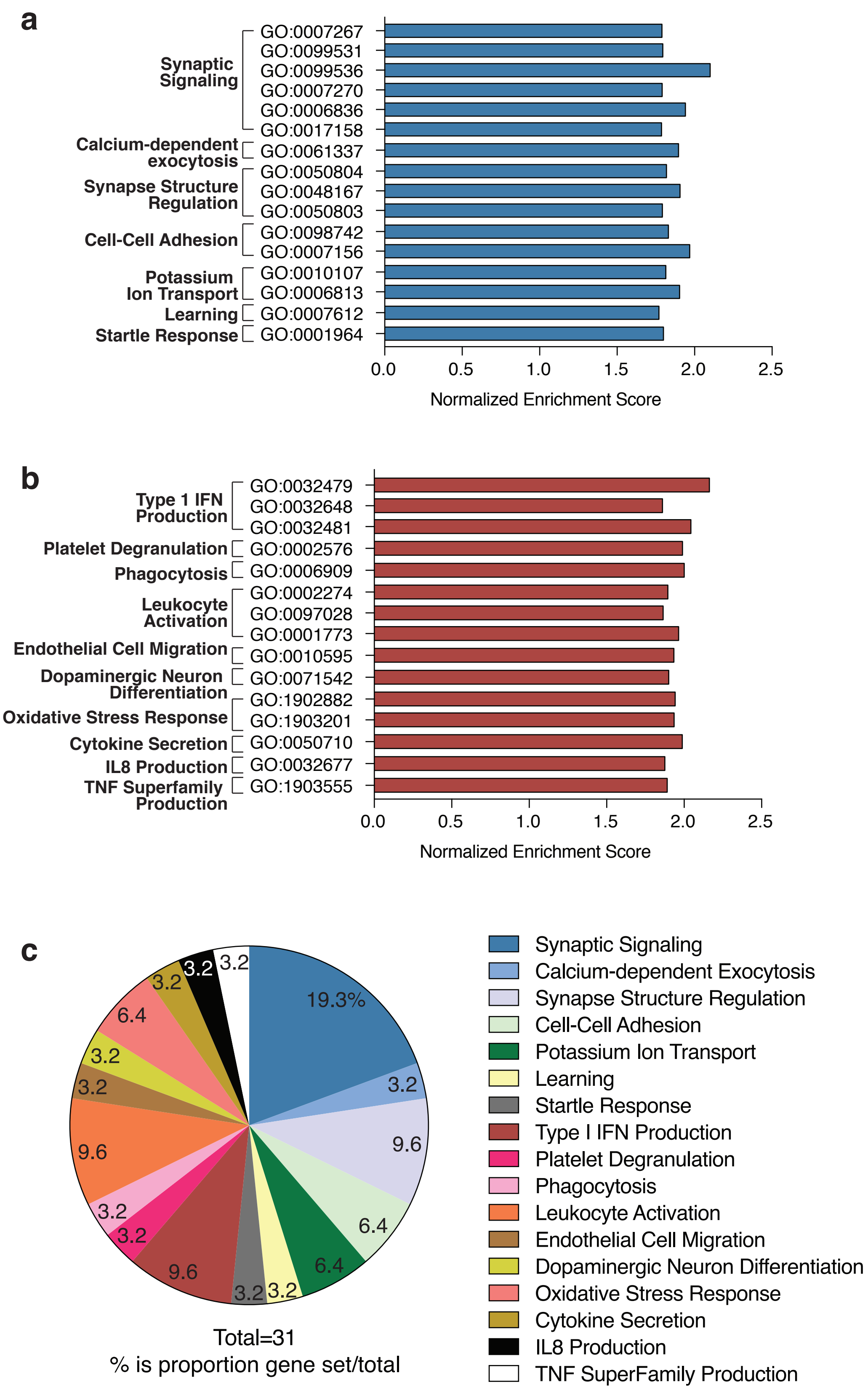

Supplementary Figure 7. ICSI of sperm incubated with corticosterone-treated DC2 EVs alter the embryonic brain transcriptome. (**a**) Following RNA sequencing analysis, total GO terms significantly enriched and (**b**) decreased in Cort^{EV} E12.5 brains determined by GSEA were grouped under parent terms. (N=6 embryos/EV treatment, FDR< 0.05). The top three significant clusters of GO terms enriched in EV^{Cort} E12.5 brains are presented in Figure 3d. (**c**) Proportion of significant child GO terms collapsed into parent terms, showing that Synaptic Signaling (19.3%) encompasses the majority of significantly altered gene sets in the E12.5 brain by ICSI of sperm incubated with EVs secreted 8-days following corticosterone treatment of DC2 cells.

Supplementary Figure 8

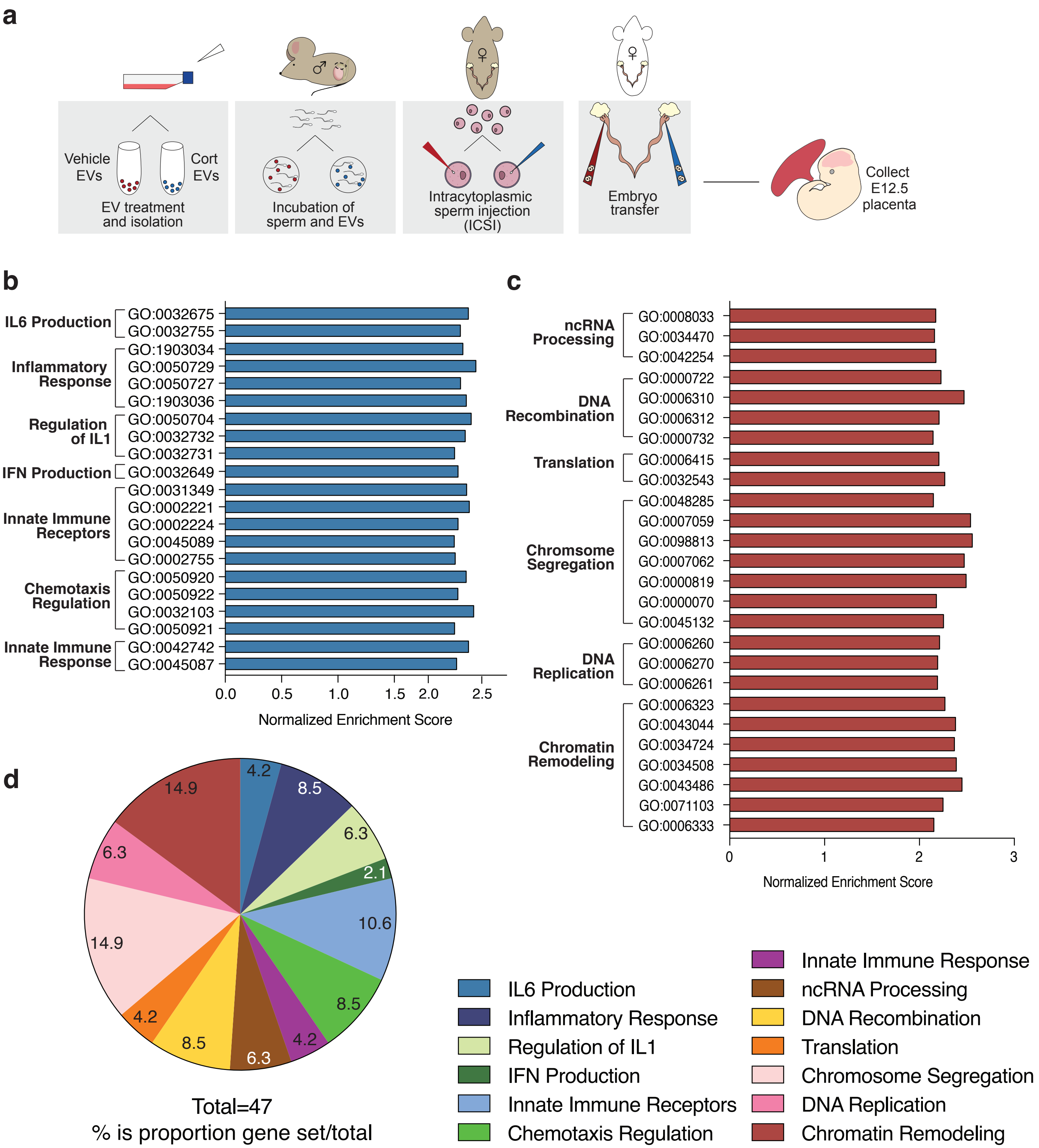

Supplementary Figure 8. ICSI of sperm incubated with corticosterone-treated DC2 EVs alters the placenta transcriptome. (**a**) To assess a causal relationship between the significant changes to secreted EV bioactive cargo and intergenerational transmission, we utilized the assisted reproductive technology, ICSI, to inject caput epididymal sperm from naïve adult male mice. Prior to injection, sperm samples were divided into two pools and briefly incubated with secreted EVs isolated 8 days post vehicle or corticosterone treatment (EV^{Veh} sperm or EV^{Cort} sperm). Sperm were then microinjected into super-ovulated oocytes obtained from the same donor females. Cleaved 2-cell zygotes from both EV-treatment groups were then transferred into the designated right or left side of the same naïve foster females, and changes to offspring neurodevelopment were assessed at mid-gestation (E12.5). Therefore, sperm, oocytes and intrauterine environments for offspring development were the same for both treatment groups, with the only difference being the EV population the sperm were incubated with prior to ICSI. Resulting placentas were collected at E12.5 for transcriptional analysis. GO terms, clustered under parent group terms to reduce redundancy, were (**b**) enriched and (**c**) decreased in $Cort^{EV}$ placentas compared to Veh^{EV} placentas (N=6 placentas/EV treatment, FDR < 0.05), showing caput epididymal epithelial cell-secreted EVs can impact placental regulation/function as well as embryo development. (**d**) Pie chart showing proportion of significantly enriched child gene sets under each parent term in total significant gene sets, showing Chromatin Remodeling and Chromosome Segregation encompass the majority of gene sets altered in the E12.5 placenta by ICSI of Cort DC2 EVs.

Supplementary Figure 9

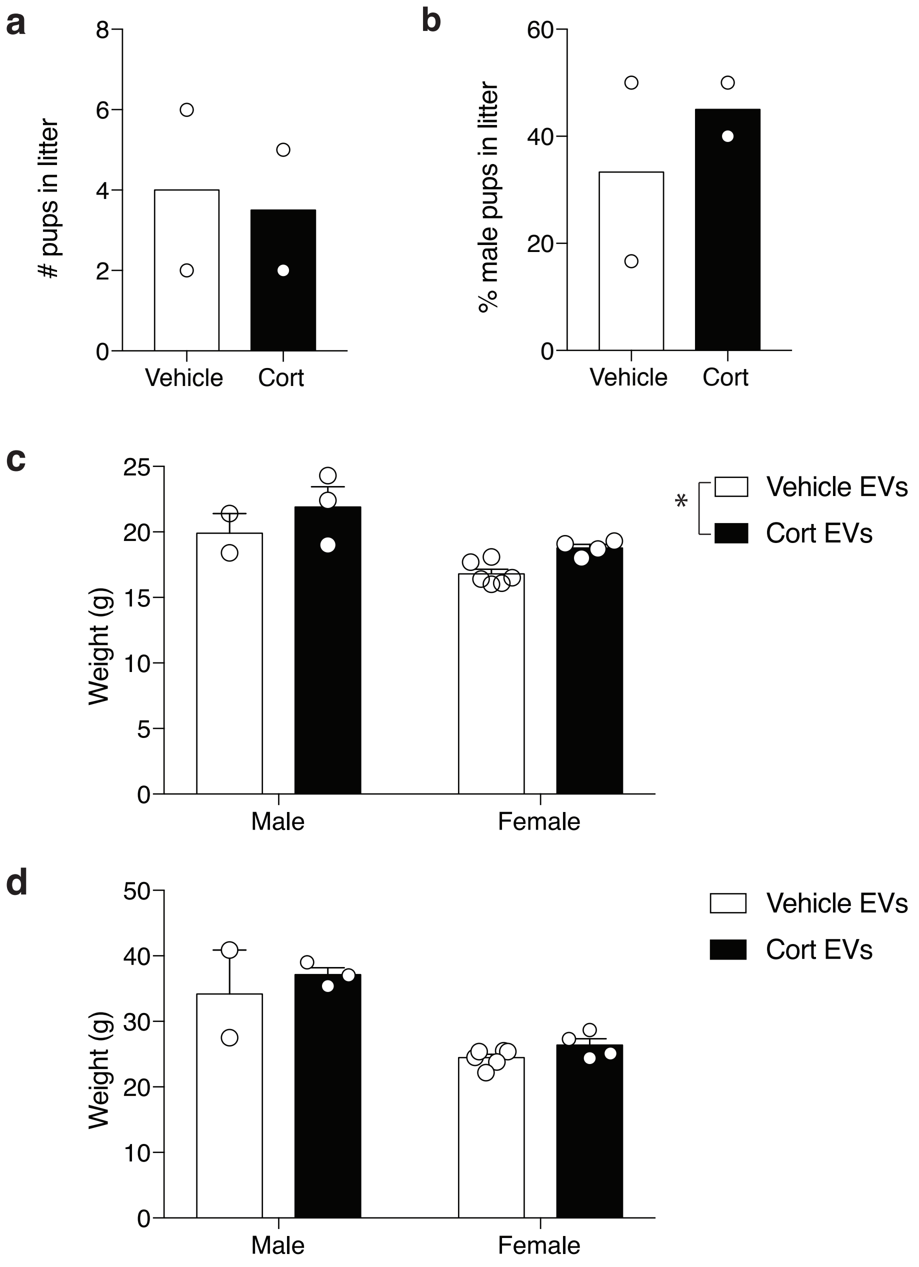

Supplementary Figure 9. ICSI of sperm incubated with corticosterone-treated DC2 EVs produce offspring with normal litter characteristics but altered physiological outcomes. (a) Litter sizes and (**b**) sex ratios produced from ICSI of sperm incubated with vehicle- or corticosterone-treated EVs, with average values indicated (N=2 litters/EV treatment). (**c**) There was a significant effect of both EV treatment and sex on body weights at 4 weeks (two-way ANOVA, main effect of sex ($F(1, 11) = 14.34$, $p = 0.003$), main effect of EV treatment ($F(1, 11) = 5.845$, $*p = 0.0341$). N=2-3 males and 4-5 females/EV treatment. (**d**) Adults weights were no longer different in both male and female offspring at 15 weeks of age (two-way ANOVA, main effect of sex ($F(1, 11) = 31.73$, $p = 0.0002$), effect of EV treatment ($F(1, 11) = 1.771$, $p = 0.2102$). N=2 males for EV^{Veh}, 3 males for EV^{Cort}, 5 females for EV^{Veh} and 4 females for EV^{Cort}. Error bars represent mean ± SEM.
