# Supplementary material for: Reproductive tract extracellular vesicles are sufficient to transmit intergenerational stress and program neurodevelopment
Source: Nat Commun. 2020 Mar 20;11:1499. doi: 10.1038/s41467-020-15305-w (PMC7083921; doi:10.1038/s41467-020-15305-w)
Supplement: Supplementary file 2 — Description of Additional Supplementary Files [file 41467_2020_15305_MOESM2_ESM.pdf]

Description of Additional Supplementary Files

File Name: Supplementary Data 1
Description: Results table from DEseq analysis of sperm miRNA

File Name: Supplementary Data 2
Description: Statistics for qRT-PCR validation of sperm and EV RNAsequencing

File Name: Supplementary Data 3
Description: Baseline demographics and assessments including ACE questionnaire and STAI inventory for all subjects in human cohort

File Name: Supplementary Data 4
Description: Data for raw caput epididymal histone mass spectrometry data

File Name: Supplementary Data 5
Description: Results table from DEseq analysis of DC2 EV miRNA

File Name: Supplementary Data 6
Description: Data for raw DC2 EV protein mass spectrometry data

File Name: Supplementary Data 7
Description: GO terms for Figure 3e, E12.5 offspring brains from ICSI of sperm incubated with EVs collected from DC2 cells following treatment

File Name: Supplementary Data 8
Description: Complete result Data for GO terms in E12.5 offspring brains from ICSI of sperm incubated with EVs collected from DC2 cells following treatment

File Name: Supplementary Data 9
Description: Complete result Data for GO terms in E12.5 offspring placenta from ICSI of sperm incubated with EVs collected from DC2 cells following treatment

:
